# ZnO Nanoparticle-Infused Vaterite Coatings: A Novel Approach for Antimicrobial Titanium Implant Surfaces

**DOI:** 10.3390/jfb16030108

**Published:** 2025-03-19

**Authors:** Atiđa Selmani, Scarlett Zeiringer, Ankica Šarić, Anamarija Stanković, Aleksander Učakar, Janja Vidmar, Anže Abram, Branka Njegić Džakula, Jasminka Kontrec, Anamarija Zore, Klemen Bohinc, Eva Roblegg, Nives Matijaković Mlinarić

**Affiliations:** 1 Pharmaceutical Technology and Biopharmacy, Institute of Pharmaceutical Sciences, University of Graz, Universitätsplatz 1, 8010 Graz, Austria; atida.selmani@uni-graz.at (A.S.); scarlett.zeiringer@uni-graz.at (S.Z.); eva.roblegg@uni-graz.at (E.R.); 2 Ruđer Bošković Institute, Bijenička Cesta 54, 10000 Zagreb, Croatia; ankica.saric@irb.hr (A.Š.); bnjeg@irb.hr (B.N.D.); jasminka.kontrec@irb.hr (J.K.); 3 Department of Chemistry, University of Osijek, Ulica Cara Hadrijana 8/A, 31000 Osijek, Croatia; astankovic@kemija.unios.hr; 4 Jožef Stefan Institute, Jamova Cesta 39, 1000 Ljubljana, Sloveniajanja.vidmar@ijs.si (J.V.); anze.abram@ijs.si (A.A.); 5 Faculty of Health Sciences, University of Ljubljana, Zdravstvena Pot 5, 1000 Ljubljana, Slovenia; anamarija.zore@zf.uni-lj.si (A.Z.);

**Keywords:** calcium carbonate, ZnO nanoparticles, titanium implants, antimicrobial activity, biocompatible composites

## Abstract

Loss of implant function is a common complication in orthopaedic and dental surgery. Among the primary causes of implant failure are peri-implant infections which often result in implant removal. This study demonstrates the development of a new antimicrobial titanium coating with ZnO nanoparticles of various sizes and morphologies immobilised in poly(allylamine hydrochloride) and alginate multilayers, combined with epitaxially grown vaterite crystals. The coated samples were characterised with various methods (FTIR, XRD, SEM) and surface properties were evaluated via water contact angle and surface charge measurements. Zinc ion release was quantified using ICP-MS. The antimicrobial efficacy of the coatings was tested against *Staphylococcus aureus*, *Staphylococcus epidermidis*, and *Candida albicans* while the biocompatibility was tested with preosteoblast cells (MC3T3-E1). Results demonstrated the successful preparation of a calcium carbonate/ZnO composite coating with epitaxially grown vaterite on titanium surfaces. The Zn ions released from ZnO nanoparticles dramatically influenced the morphology of vaterite where a new flower-like morphology was observed. The coated titanium surfaces exhibited robust antimicrobial activity, achieving over 90% microbial viability reduction for *Staphylococcus aureus*, *Staphylococcus epidermidis*, and *Candida albicans*. Importantly, the released Zn^2+^ concentrations remained below the cytotoxicity limit for MC3T3-E1 cells, showing potential for safe and effective implant applications.

## 1. Introduction

Peri-implant infections, directly related to implants, are one of the leading causes of poor implant integration. The formation of polymicrobial biofilms on titanium surfaces is recognised as the main cause of inflammatory processes in the tissues surrounding implants. Such processes often end with the loss of functionality of the implant, which requires its removal. Due to the increasing life expectancy of the population, a significant increase in the number of revision surgeries caused by peri-implant infections is projected, with increase estimates ranging from 42% to as much as 149% by 2040 [1]. Bacterial strains of the *Staphylococcus* genus most often cause implant infections. *Staphylococcus aureus* (*S. aureus*, 15.6%) and *Staphylococcus epidermidis* (*S. epidermidis*, 42%) together make up approximately two-thirds of the isolated pathogens [2]. In dental implants, *Candida albicans* (*C. albicans*) is also often isolated from biofilms associated with dental implant infections [3]. The prevention of peri-implant infections is of great social importance, and modern research is increasingly focusing on the development of antimicrobial implant coatings [4,5].

Currently, the development of new antibiotics faces challenges. Antibiotics can contribute to the strengthening of bacterial resistance to drugs [6,7,8]. Considering the increasing resistance of pathogenic microorganisms, there is a need for research and development of new, effective antimicrobial solutions. As a potential antimicrobial agent, metal-based nanoparticles (NPs) are attractive because they inactivate microorganisms even at low concentrations [9,10]. Zinc oxide (ZnO) is attractive for biomedical applications due to its low toxicity, biocompatibility, and diverse morphology [11,12,13,14,15], as well as excellent antibacterial properties against Gram-positive and Gram-negative bacteria [13,16,17,18,19]. The ZnO nanostructure properties such as size, shape, crystallinity, and surface charge critically influence their biological activity. The smaller NP size and larger specific surface area enhance antimicrobial effects [13,20]. The incorporation of ZnO NPs into biocompatible polyelectrolytes like poly(allylamine hydrochloride) (PAH) enables immobilisation on surfaces, minimising cytotoxicity while maintaining antimicrobial activity through controlled ion release [21]. The selection of (PAH) and alginate (ALG) for multilayer formation in titanium implant coatings is informed by their synergistic chemical and biological properties. These polyelectrolytes facilitate Layer-by-Layer (LbL) self-assembly through electrostatic interactions, allowing precise film thickness and NP incorporation control [22,23]. The positively charged PAH layers effectively bind negatively charged NPs (such as ZnO), ensuring stable immobilisation within the coating [24]. Both PAH and ALG are recognised for their biocompatibility; ALG, a natural polysaccharide, is particularly noted for its bioadhesive and osteoconductive properties, making it suitable for bone-contacting implants [25]. Additionally, PAH exhibits intrinsic antibacterial activity by disrupting bacterial membranes [26,27], which, when combined with ZnO’s antimicrobial properties, may enhance the coating’s efficacy against implant-associated infections. The multilayer structure also reinforces mechanical stability, improving adhesion and resistance to delamination, which is crucial for the durability of implant coatings [28,29]. Previous research on human and animal cells showed that the cytotoxic effect was more pronounced in the case of NPs being released from surfaces, while ions released from immobilised NPs caused very low cytotoxicity [30]. Zn is particularly interesting because, as a trace element in the human body, it causes an increase in the viability of human cells after their exposure to moderate concentrations of Zn^2+^ ions [31].

In addition to antimicrobial materials, bioactive coatings such as hydroxyapatite and calcium phosphate have been widely investigated for improving implant osseointegration [32]. Although hydroxyapatite (HA) demonstrates good biocompatibility, its slow resorption hinders bone regeneration and limits the recovery of damaged bone [33]. On the other hand, calcium carbonate (CaCO_3_) shows excellent biocompatibility and faster resorption than HA [34]. Among CaCO_3_ polymorphs, vaterite stands out as the most soluble and suitable for restorative applications [35]. While CaCO_3_ enhances osseointegration, it lacks inherent antimicrobial properties [36]. Integrating ZnO NPs with varying morphologies into CaCO_3_ coatings is a promising strategy to address this obstacle. Given the increasing interest in ZnO-based antimicrobial applications, defining the optimal preparation pathway process for innovative ZnO-based composites is an essential step for rational and cost-effective manufacturing. It is important to emphasise that incorporating ZnO NPs into materials increases their stability and improves the destruction potential for pathogens upon contact [37,38]. Additionally, ZnO and CaCO_3_ have been used in polymer composite production to enhance their mechanical properties, but their combined, synergistic effect or antimicrobial activity has not yet been investigated [39]. Furthermore, three-dimensional porous polymeric scaffolds comprised of chitosan, carboxymethylcellulose, ZnO, and CaCO_3_ proved to be bioactive, non-cytotoxic, and osteoinductive [40], but their antimicrobial characteristics were not evaluated. Most recently, amorphous CaCO_3_ NPs with incorporated magnesium, Zn^2+^, and strontium ions demonstrated good antibacterial activity against *S. aureus* and *Escherichia coli* and biocompatibility with fibroblast cells [41]. However, the method for amorphous CaCO_3_ growth directly on hard surfaces was not shown nor was the antimicrobial activity against *S. epidermidis* and *C. albicans* demonstrated.

This study aimed to develop a novel antimicrobial and biocompatible titanium coating by integrating ZnO nanoparticles (NPs) with diverse morphologies into PAH/alginate multilayers, combined with epitaxially grown CaCO_3_/vaterite crystals. While ZnO NPs have been used in titanium coatings, their combination with a bioactive vaterite layer has not been previously reported. A key innovation of this study is the morphological modification of vaterite through ZnO NPs, leading to unique flower-like crystal structures. This ZnO–vaterite interaction has not been previously explored and offers insights into ZnO-based bioactive coatings. Additionally, this work systematically evaluates ZnO morphology-dependent antimicrobial performance and explores the size-dependent effect in achieving the highest microbial viability reduction in *S. aureus*, *S. epidermidis*, and *C. albicans*.

To ensure biocompatibility, ZnO NPs were immobilised within PAH/alginate multilayers, minimising cytotoxicity while maintaining antimicrobial efficacy. Furthermore, cytotoxicity testing of different ZnO morphologies (SS, SR, BS, BR) was explored for the coating’s safety for potential clinical applications.

The findings of this study demonstrate the successful preparation of a CaCO_3_/ZnO composite coating with epitaxially grown vaterite crystals. The morphology and precipitation of vaterite were significantly altered on the titanium plates coated with ZnO NPs, demonstrating their influence on these properties. The coated titanium surfaces exhibited robust antimicrobial activity, achieving a microbial viability reduction of over 90% for *S. aureus*, *S. epidermidis*, and *C. albicans*. Importantly, the Zn^2+^ ion concentrations remained below cytotoxicity thresholds for MC3T3-E1 cells, highlighting the coating’s potential for safe and effective implant applications. By integrating antimicrobial, bioactive, and biocompatible properties, this study introduces a pioneering ZnO–vaterite composite coating, offering a promising strategy for infection-resistant and osseointegrative titanium implants.

## 2. Materials and Methods

### 2.1. Nanoparticle Preparation and Characterisation

Zinc acetylacetonate monohydrate (Zn(C_5_H_7_O_2_)_2_·H_2_O; 96%; Alfa Aeser^®^, Karlsruhe, Germany), zinc acetate dihydrate, (Zn(CH_3_CO_2_)_2_ ∙ 2H_2_O, Sigma-Aldrich, Schnelldorf, Germany), monoethanolamine (C_2_H_7_NO; 99%, Sigma-Aldrich, Schnelldorf, Germany), sodium hydroxide (NaOH, Sigma-Aldrich, Schnelldorf, Germany), 1-pentanol (CH_3_(CH_2_)_4_OH, Sigma Aldrich, Schnelldorf, Germany), and absolute ethanol (C_2_H_5_OH, 99.9%, J. T. Baker; Deventer, The Netherlands) were used for the preparation of ZnO samples with different sizes and morphology.

The solvothermal synthesis of ZnO NPs (smaller-sized spheres, SS ZnO) was carried out considering our previously reported procedure [42] and using a similar synthesis strategy. The ZnO sample was synthesised by adding Zn(C_5_H_7_O_2_)_2_·H_2_O (0.5 g) to an ethanolic solution of monoethanolamine (30 mL) and adjusting the molar ratio of monoethanolamine/Zn(C_5_H_7_O_2_)_2_·H_2_O to 2:1. The transparent precursor solutions prepared in this way were autoclaved at 170 °C for 24 h. A 50 mL Teflon-lined stainless-steel autoclave was used for this purpose. After autoclaving, the obtained precipitates were centrifuged, washed several times with ethanol, and then dried overnight in a vacuum at room temperature.

The solvothermal synthesis of ZnO NPs (smaller-sized rods, SR ZnO) was carried out considering our previously reported procedure [43]. In a typical synthetic procedure, zinc acetylacetonate monohydrate (Zn(C_5_H_7_O_2_)_2_·H_2_O) in predetermined amounts of 0.4 g was suspended in 30 mL of 1-pentanol. Then, the prepared transparent precursor solution was autoclaved at 170 °C for 4 h. After autoclaving, the obtained precipitates were centrifuged, washed several times with ethanol, and then dried overnight in a vacuum at room temperature.

For the preparation of bigger-sized spheres (BS ZnO) and bigger-sized rods (BR ZnO), a solution of Zn(CH_3_CO_2_)_2_ (*c* = 0.1 mol L^−1^) was prepared in a 5:1 water–ethanol mixture. Synthesis was conducted following a previously reported procedure [22] by adding 50 mL of NaOH to 150 mL of the Zn(CH_3_CO_2_)_2_ solution. For the BS ZnO, the Zn(CH_3_CO_2_)_2_ suspension was mixed with 1.0 mol L^−1^ NaOH, heated to 70 °C and continuously magnetically stirred (300 rpm) for 15 min. The BR ZnO was synthesized with the addition of 5.0 mol L^−1^ NaOH, heated to 85 °C and continuously magnetically stirred (300 rpm) for two hours. The obtained precipitates were centrifuged, washed several times with water, and then dried at 100 °C for two hours.

The PXRD pattern of the ZnO sample was recorded at room temperature using an Italstructures X-ray powder diffractometer (APD 2000, Cu-Kα radiation, graphite monochromator, scintillation detector, GNR, Novara, Italy). The morphology of the ZnO NPs was examined using a Schottky field emission scanning electron microscope (FE-SEM) Jeol JSM-7600F and JSM-7000F (Jeol Ltd., Tokyo, Japan). NPs’ size distribution was determined from the SEM images with ImageJ 2 Software(LOCI, University of Wisconsin, Madison, WI, USA). A powder X-ray diffraction (PXRD) analysis confirmed the composition of ZnO NPs. PXRD diffractograms were obtained on a PANalytical Aeris Research Diffractometer (Malvern PANalytical, Malvern, UK) and Italstructures X-ray powder diffractometer APD 2000 (GNR, Novara, Italy) with a Cu-target tube, a step size of 0.0131°, and 1 s per step in the 2*θ* range of 20–80° in Bragg–Brentano geometry. PANalytical High Score Plus 3.0 software (Malvern PANalytical, Malvern, UK) was used to analyse the diffraction patterns.

### 2.2. Titanium Coating

Titanium plates (foil, thickness 0.127 mm, ≥99.99% trace metals basis) were obtained from Sigma-Aldrich, Germany. A titanium oxide layer was produced to activate the titanium surface. For that, the 10 mm × 10 mm plates were incubated in hydrogen peroxide (H_2_O_2_, 30%, Kemika, Zagreb, Croatia) overnight. Following the incubation, the plates were washed with water and dried at 100 °C for two hours. The plates were then incubated in NaOH (1 mol dm^−3^) for one hour, washed with water, and dried for two hours at 100 °C.

**Scheme 1:**
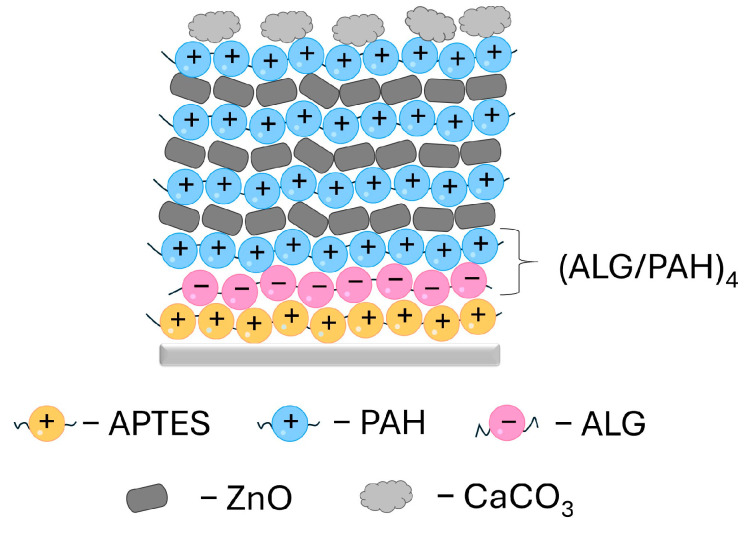
Multilayer coating prepared on the titanium surface (light grey line). The coating procedure was started with the initial layer of APTES (yellow spheres) followed by alternating 4 layers of PAH/ALG. ZnO nanoparticles (dark grey shapes) were applied between the last four PAH layers. Calcium carbonate was precipitated as the last layer (light grey shapes). APTES—(3-Aminopropyl)triethoxysilane, ALG—alginate, PAH—poly(allylamine hydrochloride).

To ensure better surface coverage with the multilayer coating, the titanium plates were pretreated with (3-Aminopropyl)triethoxysilane (APTES) 6% prepared in absolute ethanol. The multilayer was produced with poly(allylamine hydrochloride) (PAH, average *M*_w_~17,500) and alginate (ALG, Alginic acid sodium salt, powder, CAS Number: 9005-38-3), all purchased from Sigma Aldrich, Schnelldorf, Germany. The PAH/ALG multilayer was fabricated using an LbL (Layer-by-Layer) method (Scheme 1). The APTES-coated plates were subjected to multilayer deposition of (ALG/PAH)n, where the number of bilayers is represented by n, through alternate immersion in ALG (3.0 g dm^−3^, pH 7.5) and PAH (3.0 g dm^−3^, pH 7.5) solutions for 15 min. In this study, four ALG/PAH layers, n = 4, were applied before plate incubation in a ZnO NP suspension (1500 g dm^−3^) between the last four PAH layers (Scheme 1). The surface underwent coating with three layers of ZnO NP suspension with an incubation time of 20 min. The coating process was finished with a final layer of PAH. After each layer deposition, the surface was rinsed with deionized water and dried with warm air.

For the CaCO_3_ epitaxial growth, titanium plates with polyelectrolyte layers with and without NPs were used. The coated plates were firstly covered with a 100 mL, 0.04 mol dm^−3^ solution of sodium bicarbonate (NaHCO_3_, Sigma Aldrich, Schnelldorf, Germany) containing 0.1 mol dm^−3^ sodium chloride (NaCl, Sigma Aldric, Schnelldorf, Germany) and 10 ppm poly-*L*-aspartic acid (pAsp, CAS#34345-47-6; 100 L-aspartic Acid Repeating Units, *M*_w_ = 14,000 Da, Alamanda polymers, Huntsville, AL, USA). Secondly, 100 mL of 0.04 mol dm^−3^ calcium chloride (CaCl_2_·2H_2_O, Sigma Aldrich, Schnelldorf, Germany) solution containing 0.1 mol dm^−3^ NaCl was slowly added to the NaHCO_3_/NaCl/pAsp solution and gently mixed. The final concentrations after mixing were *c*(CaCl_2_) = *c*(NaHCO_3_) = 0.02 mol dm^−3^, *c*(NaCl) = 0.1 mol dm^−3^, *γ*(pAsp) = 5 ppm. The obtained solution was left for 7 days at 25 °C. The plates overgrown with CaCO_3_ crystals were washed with water and dried at 100 °C for two hours. The surrounding CaCO_3_ crystals, not precipitated on the titanium plates, were scraped off the glass surface of the laboratory beaker, washed with water, and dried for two hours at 100 °C for further analysis.

### 2.3. Characterisation of the Coated Titanium Plates

The FTIR spectra of the titanium plates coated with PAH/ALG multilayers embedded with ZnO and precipitated CaCO_3_ were measured on a Bruker Tensor II with an ATR module equipped with a diamond crystal. Each FTIR spectrum was obtained as an average of 16 scans with a resolution of 4 cm^−1^. A PXRD analysis of the coated titanium plates and the CaCO_3_ precipitated around the plates was performed in the range of 2*θ* = 20–80° on a PANalytical Aeris Research Diffractometer (Malvern PANalytical, Malvern, UK) with CuKα radiation, a step size 0.003°, and 23.97 s per step. Data were processed using PANalytical’s proprietary software, X’Pert HighScore Plus 3.0 (Malvern PANalytical, Malvern, UK). ZnO, titanium, calcite, and vaterite were identified based on the ICDD Powder Diffraction Files 36-1451, 44-1294, 01-072-1937, and 01-072-0506, respectively. The quantitation of phases was determined using the reference intensity ratio (RIR) method [44].

The hydrophobicity of the coated titanium plates was determined by water contact angle measurement. The static contact angle between the titanium surface (10 mm × 10 mm) and water droplets was conducted on the Attension Theta tensiometer (Biolin Scientific AB, Gothenburg, Sweden) via the sessile-drop technique in quadruplicate. Briefly, a 5 µL water droplet was dropped from a needle with a 0.4 mm diameter onto the titanium plate surface, and the static contact angle was measured. The analysis of the streaming potential on the coated titanium plates was conducted in a 1 mmol dm^−3^ KCl solution on a SurPASS electrokinetic analyser (Anton Paar GmbH, Graz, Austria) at room temperature and pH ≈ 6.

For Zn^2+^ ion release studies, titanium plates were covered with 1 mL of water and incubated at room temperature for 24 h. For the total concentration of Zn on the titanium plates, the sample was incubated in a 1 mL, 0.5 mol dm^−3^ HCl solution for 24 h. The solution above the titanium plates was collected, and the released Zn^2+^ was measured on an Agilent 7900x inductively coupled plasma mass spectrometer (ICP-MS) (Agilent Technologies, Tokyo, Japan) equipped with an autosampler (SPS-4, Agilent Technologies, Tokyo, Japan), double-spray, Scott-type spray chamber made of quartz, and a glass Micromist nebulizer. Before the ICP-MS analysis, the samples that were acidified with HCl for determination of the total amount of ZnO were first mixed for 10 s using a vortex mixer (Vibromix 10, Tehtnica, Železniki, Slovenia) and subsequently diluted 50 times with 1% nitric acid (prepared from 67–69% HNO_3_, supra-pure, Carlo Erba Reagents, Cornaredo, MI, Italy). In samples immersed in deionized water and incubated for 24 h at room temperature, the concentration of Zn^2+^ ions and ZnO NPs were measured. An aliquot of the collected solution was acidified with 1 M HCl (prepared from 29–31% HCl, pico-pure, Chem-Lab NV, Zedelgem, Belgium) to dissolve ZnO NPs, and subsequently diluted 2.5 times with 1% nitric acid. Also, to separate ZnO NPs from the dissolved Zn fraction, another aliquot was subjected to ultrafiltration using 3 kDa (about 1–2 nm pore size) Amicon Ultra-4 Centrifugal Filter Units (Merck Millipore, Milford, MA, USA) and centrifuged at 9000 rpm for 60 min using a Hettich Universal 320 centrifuge (Hettich, Beverly, MA, USA). The filtrates were diluted 5 times with 1% nitric acid before the ICP-MS analysis. The quantity of released ZnO NPs from the titanium surface was obtained from the difference between the solution acidified with HCl (to dissolve ZnO NPs) and the filtered solution containing only the dissolved Zn fraction. A blank sample (i.e., ultrapure water) was also subjected to ultrafiltration to assess the concentration of Zn leached from the filter membranes (Amicon Ultra-4 Centrifugal Filter Units, Merck Millipore, Milford, MA, USA). This value was then subtracted from the Zn concentrations measured in the sample filtrates. Milli-Q water (18.2 MΩ cm) obtained from the Direct-Q 5 Ultrapure aqueous system (Merck Millipore, Milford, MA, USA) was used for all sample preparation and dilution.

### 2.4. Antimicrobial Studies

A colony of *Staphylococcus aureus* ATCC 25923 (*S. aureus*) and a colony of *Staphylococcus epidermidis* ATCC 14990 (*S. epidermidis*) were each transferred into 5 mL of brain heart infusion growth medium (Biolife Italiana, Milano, Italy). Furthermore, one colony of *Candida albicans* ATCC 36232 (*C. albicans*) was transferred into 5 mL of Sabouraud nutrient broth (Biolife Italiana, Milano, Italy). The prepared microbial suspensions were incubated overnight at 37 °C without shaking. Following the incubation, the optical density of the overnight cultures was measured at OD600 [45,46] to achieve a final concentration of 10^5^ CFU mL^−1^. The optical density of the microbial suspensions was measured in 96-well plates in triplicate on a spectrophotometer (Tecan, Mannedorf/Zürich, Switzerland) at a wavelength of 600 nm. The 10 mm × 10 mm titanium plates were sterilized under UV light and transferred into a sterile 24-well plate (n = 3). Uncoated titanium and titanium plates with CaCO_3_ grown on polyelectrolyte layers without ZnO NPs were used as control samples. The antimicrobial testing was performed by adding 100 μL of the diluted *S. aureus*, *S. epidermidis* and *C. albicans* suspension onto the titanium plate surface in triplicate and incubating at 37 °C for 24 h. After incubation, the titanium samples were twice washed with a 0.9% NaCl solution (1 mL). The washing solution was collected and used for agar spotting, after a series of dilutions. The washed titanium plates were added into a flask containing 2.5 mL of 0.9% NaCl solution, sonicated for 5 min in a sonication water bath, and vigorously vortexed at the highest speed for 15 s. To enumerate planktonic and adherent cells after a series of dilutions, 10 μL of the microbial suspensions was spotted, n = 3, on the plate count agar plates and incubated for 18 h at 37 °C.

The antimicrobial efficiency of the coating was expressed as the percentage reduction in the viable *S. aureus*, *S. epidermidis*, and *C. albicans* cells, according to Equation (1):

*P* = (1 − B/A) × 100%, (1)

from the number of viable microbial cells on the uncoated titanium plates (A) and the number of viable microbial cells on the coated titanium plates (B).

### 2.5. In Vitro Cytocompatibility Test

The cytotoxicity studies were conducted on the osteoblast precursor cell line (MC3T3-E1, ECACC, Salisbury, UK; kindly provided by Dr. Begüm Okutan, Medical University Graz). Cells were cultivated in 10 mL high-glucose Dulbecco’s modified eagle medium (DMEM) supplied with 10% foetal bovine serum, 1% penicillin/streptomycin (Gibco, Life Technologies Corporation, Painsley, UK) at 37 °C and 5% CO_2_ until they reached 80% of confluence. After media removal and rinsing with sterile phosphate-buffered saline (PBS), 1 mL of 0.25% trypsin–EDTA solution (Sigma–Aldrich, St. Louis, MO, USA) was added to the flask and incubated for 10 min at 37 °C and 5% CO_2_ to detach the cells. The detached cells were counted by the TC20 automated cell counter (Biorad, Hercules, CA, USA). Subsequently, cells were seeded in sterile 96-well plates (Thermo Fisher Scientific, Waltham, MA, USA) with a seeding density of 20,000 cells/well and incubated for 24 h at 37 °C and 5% CO_2_. After the attachment of the cells, they were washed with PBS, covered with 100 μL of the serum-free media with Zn^2+^ ions (0 < *γ*(Zn^2+^) < 20) μg mL^−1^ and SS, BS, SR, BR ZnO NPs (0 < *γ*(Zn^2+^) < 50) μg mL^−1^ (n = 6), and incubated for 4 h. Next, cells were washed with Hank’s Balanced Salt Solution (HBSS, Gibco, Life Technologies Corporation, Painsley, UK) and 20 μL of CellTiter 96^®^ (Aqueous One Solution Proliferation Assay, Promega, Madison, WI, USA), and 100 μL of HBSS was added. The plates were incubated for three hours at 37 °C and 5% CO_2_. Absorbance was measured at 490 nm using a microplate reader (CLARIOstarPlus, BMG LABTECH, Ortenberg, Germany).

### 2.6. Statistical Analysis

All experiments were conducted in triplicate if not stated otherwise in the previous sections. Statistical analysis was performed using the Tukey test (GraphPad Prism 8, La Jolla, CA, USA) and one-way and two-way analyses of variance (ANOVAs), with significance levels set at * *p* ≤ 0.0346, ** *p* = 0.0093, *** *p* < 0.0005, **** *p* < 0.0001, and ns—nonsignificant. Statistical significance was considered for the 95% confidence, i.e., a *p*-value below 0.05.

## 3. Results and Discussion

### 3.1. ZnO NP Characterisation

Among the various synthesis methods for ZnO NPs, the solvothermal method is attractive due to its simplicity and excellent control over particle size, shape, and dispersibility [43,47]. In this study, ZnO NPs were synthesized by a solvothermal synthesis using different alcoholic reaction solvents, including an ethanolic solution of monoethanolamine (SS ZnO), 1-pentanol (SR ZnO), and varying NaOH concentrations (BS ZnO and BR ZnO). ZnO can exhibit different morphologies—one-dimensional (1D), two-dimensional (2D), or three-dimensional (3D)—depending on the influence of organic additives known as “growth modifiers”. Several growth modifiers, including alcohol [43,48], plant extracts [49,50,51], sodium hydroxide [24,52,53,54], and ethanolamines (which combine the properties of amines and alcohols) [42,47,55,56,57,58] significantly alter the surface properties of ZnO particles.

**Figure 1:**
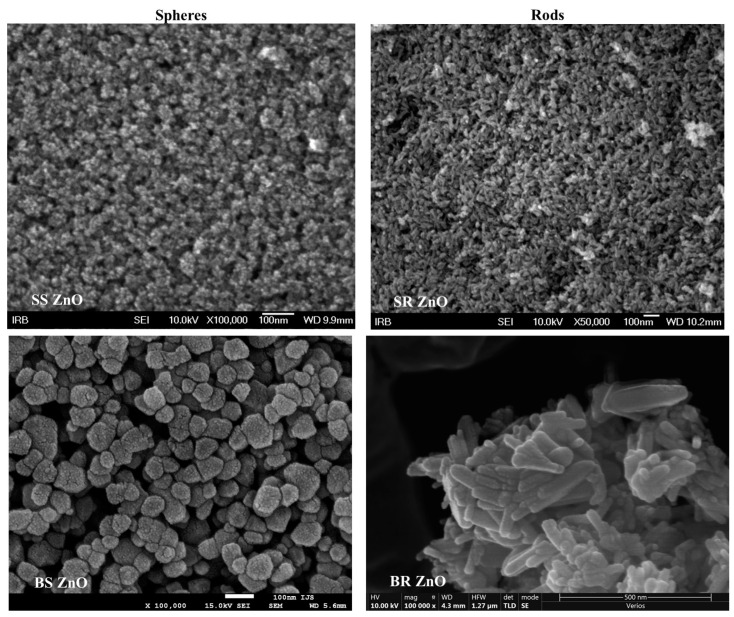
SEM images of ZnO nanoparticles with different sizes and morphology. SS ZnO and SR ZnO—smaller-sized sphere and rod-like ZnO nanoparticles, respectively; BS ZnO and BR ZnO—larger-sized sphere and rod-like ZnO nanoparticles, respectively.

To characterise the microstructure and morphology of the ZnO NPs, PXRD and SEM analyses were performed (Figure 1 and Figure S1). The PXRD patterns of ZnO NP (Figure S1) exhibited characteristic diffraction peaks at 2*θ* angles of 31.7°, 34.3°, 36.3°, 47.4°, 56.5°, 62.7°, 66.3°, 68.0°, 70.0°, 72.5°, and 76.8° corresponding to crystallographic planes (*hkl*) (100), (002), (101), (102), (110), (103), (200), (112), (201), (004), and (202), respectively. These crystallographic planes were confirmed using JCPDS card number 36-1451. The PXRD results indicated the formation of a hexagonal wurtzite ZnO structure with a P63*mc* space group symmetry. The intensity ratio of the diffraction lines was in good agreement with the intensity ratios presented in ICCD-PDF2 card 36-1451. As shown in Figure S1, the PXRD patterns of the SR ZnO sample as well as the SS ZnO sample exhibited a significant broadening of the diffraction lines, indicating the presence of very fine ZnO nanoparticles compared to the PXRD patterns of the BR and BS ZnO samples. BS and BR ZnO diffractograms exhibited much sharper and narrower peaks due to the large size of crystallites and well-ordered crystalline material [59]. Consistent with previous reports [47,60], in the prepared SS and SR samples, an X-ray diffraction size–strain analysis showed the presence of size anisotropy with significantly narrower diffraction lines along the direction <00l> in the samples ZnO obtained in 1-pentanol (SR ZnO sample) as well as in the sample obtained in the presence of monoethanolamine additive (SS ZnO sample). The obtained full width at half-maximum (FWHM) value of diffraction line 002 appeared to be significantly narrower compared to the FWHM values of the neighbouring diffraction lines 100 and 101, indicating the presence of size anisotropy with bigger crystallites in the direction of the *c*-axis (25 nm for SR ZnO and 10 nm for SS ZnO) of the zincite lattice compared to the direction perpendicular to the *c*-axis (13 nm for SR ZnO and 7 nm for SS ZnO). The results indicated the presence of a size anisotropy with a significantly larger crystal growth in the direction parallel to the *c*-axis compared to the directions perpendicular to the *c*-axis.

The morphology of ZnO NPs was further examined using FE-SEM (Figure 1). SS ZnO NPs exhibited rounded particles with an average size distribution of (6 ± 1) nm (Figures S2 and S3a). SR ZnO NPs displayed a rod-like morphology with an average size of (24 ± 4) nm (Figure S3b). BS ZnO NPs, synthesised with a lower NaOH concentration (1 mol dm^−3^), exhibited a spherical morphology with a broad size distribution (62 ± 17) nm (Figure S3c). In contrast, BR ZnO NPs, synthesised with a higher NaOH concentration (5 mol dm^−3^), had a rod-like morphology with an average size of (88 ± 27) nm (Figure S3d).

The size and morphology of ZnO NPs are governed by precursor selection, reaction temperature, solvent composition, pH, and surfactant/additive presence [61,62,63,64,65,66]. Due to the complexity of ZnO formation mechanisms, experimental and theoretical approaches [43,60,67] have been used to characterise ZnO nano- and microstructures synthesised under varying conditions, particularly focusing on solvent effects and additive presence. The size, morphology, properties, and antimicrobial performance of ZnO particles can be tailored through various synthesis methods. The resulting ZnO morphologies include smaller spheres (SS ZnO), smaller rods (SR ZnO), larger spheres (BS ZnO), and larger rods (BR ZnO), as shown in the SEM images of Figure 1. The shape and aspect ratio of ZnO nanostructures likely results from a balance between intrinsic structural factors and experimental conditions, particularly solvent effects (ethanol, 1-pentanol, aqueous NaOH). The physical properties of solvents and additives (polarity, viscosity, etc.) significantly influence ZnO particle formation. This mechanism was explored through detailed quantum chemical simulations of ZnO surface interactions using density functional theory (DFT) [43,60,67]. The combined experimental and theoretical approach provided deeper insight into ZnO formation and growth mechanisms while enabling better control over size and morphology. Experimental findings, supported by DFT results, were used to predict ZnO particle formation [43,60,67].

The formation process involves several sequential stages: prenucleation, nucleation, crystal growth, and possible aggregation, all influenced by solvent molecules of different polarities and sizes. Solvent properties such as polarity and viscosity significantly affect ZnO nucleation and growth, as demonstrated by density functional theory (DFT) calculations, which has revealed that ZnO nucleation and growth are thermodynamically governed by interface–alcohol interactions [43]. The alcohol acts as a solvent, reactant, and controlling agent for particle growth. The solvothermal synthesis of ZnO NPs in pure alcohols enables the precise control of reaction rates and provides insight into the chemical mechanisms governing their formation. Based on microstructural and theoretical studies, the nucleation and preferential growth mechanism of SR ZnO NPs in 1-pentanol was proposed [43]. The hierarchical crystal growth along the *c*-axis into a rod-like hexagonal structure is largely dictated by prenucleation intermediates and their reactivity, which influence subsequent nucleation and crystal growth steps. DFT calculations reveal that ZnO nucleation and growth in alcohols of different sizes and polarities are driven by various interactions, including van der Waals forces, coordinate bonding, hydrogen bonding, and screening effects, likely acting synergistically. Strong O–H∙∙∙O hydrogen bonds dominate in polar media, while weaker van der Waals forces prevail in nonpolar environments. Theoretical simulations of interface–solvent interactions using (ZnO)_36_−CH_3_(CH_2_)_n_OH (n ≤ 7) suggest that ZnO growth rates along the *c*-axis are primarily controlled by interface–alcohol interactions, with the most stable ZnO–alcohol structures exhibiting hydrogen bonding more than twice as strong as Zn–O coordination bonding. Thermodynamically, ZnO nucleation and growth occur through a sequence of energy barriers associated with prenucleation, nucleation, and subsequent growth processes [43].

The synthesis of SS ZnO NPs follows a nonhydrolytic route, where surfactants in the reaction mixture provide precise control over crystallite size, shape, and dispersibility. Monoethanolamine, acting as a growth modifier, influences ZnO morphology by combining the properties of amines and alcohols [60]. The nucleation process begins with the formation of primary ZnO NPs, which subsequently aggregate into well-defined nanospheres. Growth control is primarily achieved through the strong adsorption of monoethanolamine’s flexible hydroxyl chains onto ZnO nuclei, limiting spontaneous nucleation and slowing primary NP growth. Additionally, ethanolamine adsorption alters surface energy, reducing anisotropic growth, and resulting in loosely assembled fine spherical ZnO NPs. The strong affinity of monoethanolamine and ethanol for the (ZnO)_36_ surface ensures high coverage, restricting particle size while promoting interparticle attraction via hydrogen bonding. The presence of weaker N−H∙∙∙O hydrogen bonds between ethanolamine’s amine groups and ZnO further facilitates spherical aggregation [60]. Experimental conditions, including solvent polarity and additives, play a crucial role in tuning ZnO NP size and morphology by balancing electrostatic interactions and preventing excessive aggregation.

Hierarchical crystal growth along the *c*-axis is observed in the aqueous NaOH solution during the synthesis of BR ZnO, a process elucidated through combined experimental and theoretical studies [67]. The preferential *c*-axis growth is driven by interactions between water molecules, hydroxyl groups from NaOH, and the ZnO surface. DFT calculations indicate that hydrogen transfer from water to oxygen in the (ZnO)_36_ cluster plays a crucial role, with the spontaneous deprotonation of H_2_O molecules and direct OH^−^ attack on ZnO surfaces facilitating rod-like growth. Conversely, BS ZnO particles favour spherical aggregation due to their lower surface area, while terminal Zn^2+^ and O^2−^ molecules along the (002) crystal plane strongly interact with dislocated polar H_2_O molecules (OH^−^, H^+^), further influencing the crystallisation pathway. The synthesised ZnO NPs with varying morphologies and sizes were subsequently used to develop antimicrobial and bioactive titanium coatings.

### 3.2. ZnO/CaCO_3_ Composite on Titanium Surface

Prior to coating, the titanium surfaces were activated using H_2_O_2_ treatment. The untreated titanium plates showed no significant presence of an oxide layer (Figure S4a). However, following surface activation with H_2_O_2_, a titanium oxide layer was formed, as confirmed by a broad peak around 644 cm^−1^ in the FTIR spectra (Figure S4a), corresponding to titanium dioxide [68]. A morphological analysis revealed pronounced surface roughness with micro-cracks after treatment (Figure S4b). These changes are consistent with previous findings, demonstrating that 24 h H_2_O_2_ treatment alters surface topography, increases roughness, and modifies oxide thickness, which in turn enhances protein adsorption and may promote osteoblast attachment and implant osseointegration [69].

Following titanium surface pretreatment, a positively charged APTES layer was applied due to its strong bonding capability with titanium surfaces and its role as a linking agent [70,71,72]. The presence of APTES was confirmed by silicon atom detection in the EDS spectra of the titanium surfaces (Figures S5 and S6, Tables S1 and S2).

**Figure 2:**
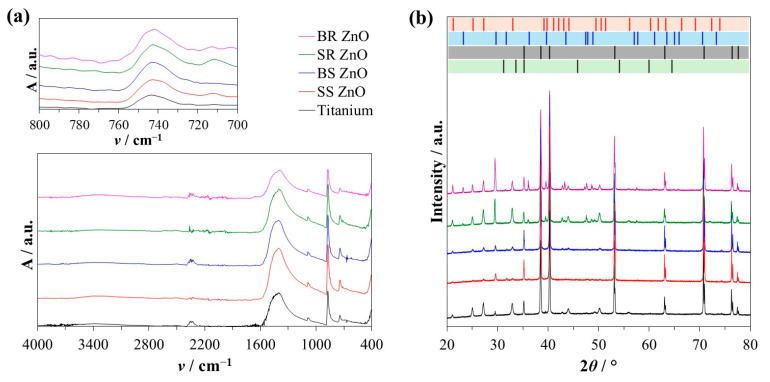
Characterisation of the titanium plate surfaces after epitaxial growth experiments. (**a**) Representative Fourier-transformed infrared spectra showing the sample absorbance (*A*) at specific wavenumber (*ν*/cm^−1^) and (**b**) X-ray diffraction intensity of the sample at specific 2*θ* angles. The standard diffraction reflections of vaterite (red bars), calcite (blue bars), ZnO (green bars), and titanium (black bars) are shown for comparison. ZnO nanoparticles with different sizes and morphology were used for surface treatment. SS ZnO and SR ZnO—smaller-sized sphere and rod-like ZnO nanoparticles, respectively. BS ZnO and BR ZnO—larger-sized sphere and rod-like ZnO nanoparticles, respectively.

After treating the titanium plates with polyelectrolyte layers containing ZnO NPs and epitaxially grown CaCO_3_ crystals, the coating composition was analysed using FTIR and PXRD (Figure 2). The spectroscopic analysis of titanium surfaces after seven days of CaCO_3_ growth revealed characteristic peaks corresponding to vaterite and calcite crystals (Figure 2a). Specifically, the presence of vaterite was confirmed by a peak at 745 cm^−1^, while calcite was identified by a peak at 713 cm^−1^. Both vibrations correspond to the *ν*_4_ O−C−O bending (in-plane deformation) mode (Table S3) [73].

A series of experiments (Table S4) were conducted to determine the optimal initial conditions for epitaxial vaterite growth. Results showed that an equimolar Ca/HCO_3_ system at 0.02 mol dm^−3^, combined with 5 ppm pAsp and 0.1 mol dm^−3^ NaCl, produced the highest amounts of stable vaterite directly on the titanium surface. In this system, the intensity of vaterite peaks decreased, indicating that Zn^2+^ ions inhibited vaterite growth while promoting calcite crystal formation.

**Figure 3:**
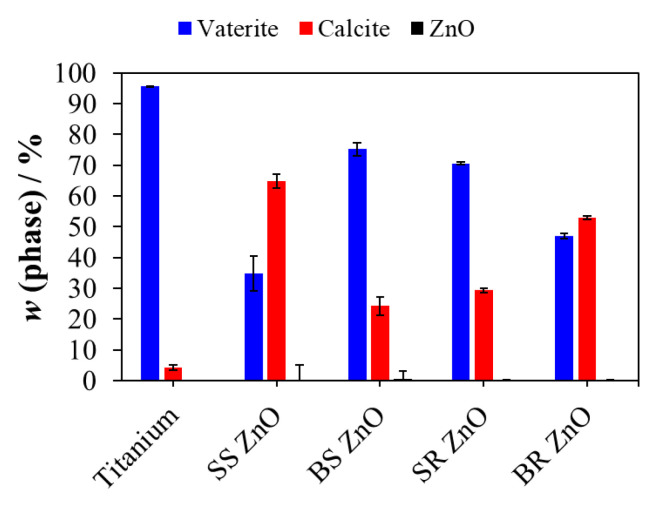
The phase composition of the surface of titanium plates after epitaxial growth experiments. ZnO nanoparticles with different sizes and morphology were used for surface treatment. SS ZnO and SR ZnO—smaller-sized sphere and rod-like ZnO nanoparticles, respectively. BS ZnO and BR ZnO—larger-sized sphere and rod-like ZnO nanoparticles, respectively.

A PXRD analysis was conducted to analyse the surface composition (Figure 2b and Figure 3). Specific diffractions of vaterite crystals were observed at 2*θ* angles of 20.8°, 24.9°, 27.1°, 32.7°, 40.7°, 42.7°, 43.8°, 49.1°, 50.0°, 55.8°, 62.6°, and 72.0° corresponding to crystallographic planes (*hkl*) (002), (020), (021), (022), (023), (004), (130), (202), (114), (222), (134), and (135), respectively, validated by JCPDS card number 01-072-0506 (Figure 2b, red bars). Also, the highest diffractions of calcite were observed at 2*θ* angles of 29.3°, 39.3°, and 47.5°, corresponding to crystallographic planes (*hkl*) (104), (113), and (018), respectively, validated by JCPDS card number 01-072-1937 (Figure 2b, blue bars).

Since the PXRD measurements were conducted directly on the titanium plates, the titanium-specific peaks were observed at 2*θ* angles of 35.2°, 38.5°, 40.3°, 53.1°, 63.0°, 70.8°, 76.3°, and 77.5°, corresponding to crystallographic planes (*hkl*) (100), (002), (101), (102), (110), (103), (112), and (201), respectively, validated by JCPDS card number 44-1294 (Figure 2b, black bars). The titanium content was excluded from the PXRD composition analysis, and the phase composition of vaterite, calcite, and ZnO is shown in Figure 3. In all prepared samples, the ZnO content remained below 0.3 wt%. An EDS analysis further corroborated these findings (Figures S5 and S6, Tables S1 and S2), albeit with deviations depending on the location of the EDS analysis. The relative amount of ZnO in the multilayers was low compared to the CaCO_3_ phases.

As shown in Figure 3, on titanium surfaces without ZnO NPs, epitaxially grown CaCO_3_ consisted of more than 95 wt% vaterite. Despite a seven-day precipitation period, an exceptionally high amount of vaterite was obtained, indicating that pAsp and high NaCl concentrations stabilised vaterite formation and prevented its transformation into the more stable calcite phase [74,75,76].

However, the presence of ZnO NPs on titanium surfaces altered the polymorph composition, reducing the vaterite content. The lowest vaterite fraction, (35.8 ± 5.7) wt% (Figure 3), was observed on titanium plates coated with SS ZnO NPs. Previous research has reported conflicting findings, with some studies indicating an absence of vaterite formation in spontaneous precipitation systems [77,78,79], while others suggest that ZnO and Zn^2+^ ions promote vaterite formation [80,81]. Some studies have further shown that at temperatures above 40 °C, Zn^2+^ ions inhibit calcite formation and favour the precipitation of metastable aragonite [82]. These discrepancies suggest that factors such as solution composition, supersaturation levels, additives, and substrate type significantly influence CaCO_3_ polymorph formation.

Our study showed that under the experimental conditions used, ZnO NP-treated titanium surfaces preferentially inhibited vaterite precipitation more than calcite precipitation (Figure 3). It was hypothesised that during CaCO_3_ growth, Zn^2+^ ions were released from the ZnO NP-treated titanium surfaces, thereby inhibiting vaterite formation. To confirm this, CaCO_3_ crystals were scraped from the glass walls of the beaker surrounding the titanium plates. The results (Figures S7 and S8) showed that in all systems, more than 95 wt% of vaterite was formed, indicating that Zn^2+^ ions exerted a localised effect on CaCO_3_ precipitated directly on ZnO NP-treated surfaces.

**Figure 4:**
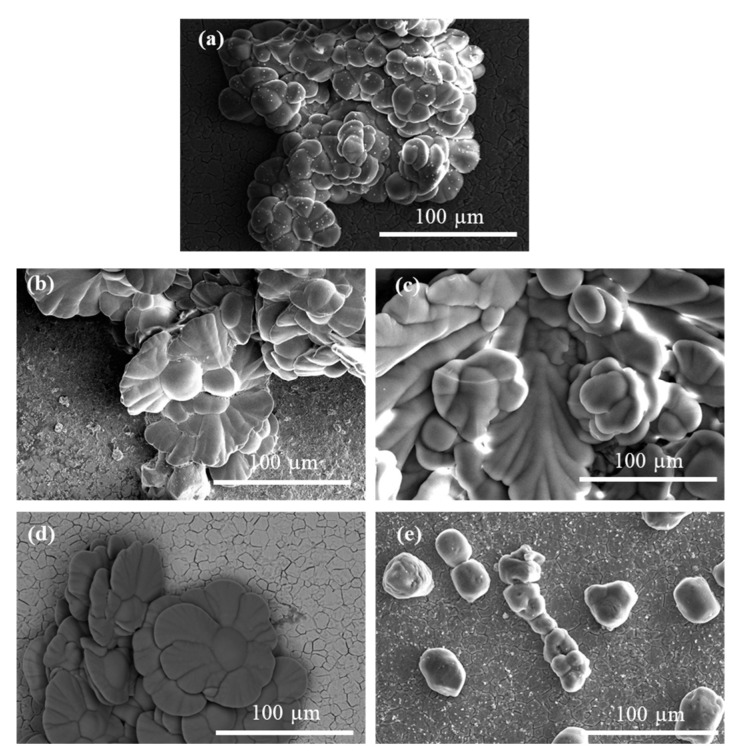
SEM images of titanium plate surfaces after calcium carbonate epitaxial growth experiments on titanium coated with PAH/ALG multilayers (**a**) without ZnO and PAH/ALG multilayers containing four types of ZnO nanoparticles: SS ZnO (**b**) and SR ZnO (**c**)—smaller sized sphere- and rod-like ZnO nanoparticles, respectively, BS ZnO (**d**) and BR ZnO (**e**)—larger sized sphere- and rod-like ZnO nanoparticles, respectively, ALG—alginate, PAH—poly(allylamine hydrochloride).

Furthermore, the morphology and surface coverage of CaCO_3_ crystals on titanium plates were analysed using SEM (Figure 4). On titanium plates without ZnO NPs, vaterite crystals exhibited an aggregated, rounded, globular morphology (Figure 4a). Similar spherical vaterite particles have previously been observed in the presence of pAsp [74,76]. Due to the slow crystal growth directly on the titanium surface, individual spherical vaterite micro-aggregates overgrew and merged, forming larger globular structures.

The vaterite crystals formed on SS ZnO- and BS ZnO-treated surfaces exhibited a closely packed, petal-like morphology, with each petal appearing smooth, curved, and relatively thin (Figure 4b,d). Overlapping petals forming dense, flower-like aggregates, were obtained in the presence of 2-naphthaleneacetic acid and ethylene glycol [83]; however, this morphology was different than the one shown here (Figure 4b,d). Vaterite crystals formed in the presence of ZnO NPs and polydimethylsiloxane displayed different flower-like morphology, lacking smooth surfaces or petal-like structures [81]. The combined effect of Zn^2+^ ions and pAsp, which has not been previously reported in the literature on vaterite formation and its morphological characteristics, resulted in this unique flower-like morphology.

On SR ZnO-treated surfaces, vaterite crystals exhibited a distinctive morphology, characterised by a combination of elongated, petal-like structures and broader, rounded surfaces, with an overall increase in crystal size (Figure 4c). These elongated petals featured smooth edges and a curved form.

The vaterite crystals on BR ZnO surfaces displayed a rounded, pillar-like morphology with a relatively smooth surface texture (Figure 4e). Aggregates of these pillar-like structures were observed to form chain-like arrangements. This unique chaining pattern may indicate specific growth dynamics or aggregation processes during crystallisation. Lower surface coverage with crystals (Figure S9, showing a bigger titanium surface area) was observed on surfaces coated with BR ZnO NPs. The application of ZnO NPs limited surface coverage with crystals, consistent with previous research demonstrating Zn^2+^ anti-scaling properties [77,82]. These results suggest that ZnO NPs of different morphologies influence the formation of crystals with distinct morphological characteristics.

Calcite crystals formed on untreated titanium surfaces exhibited continuous growth with sharp macro-steps (Figure S9a). In contrast, calcite crystals grown on ZnO NP-treated surfaces had smoother edges and exhibited less orderly and regular crystal growth compared to those on untreated surfaces (Figure S9b–e).

**Figure 5:**
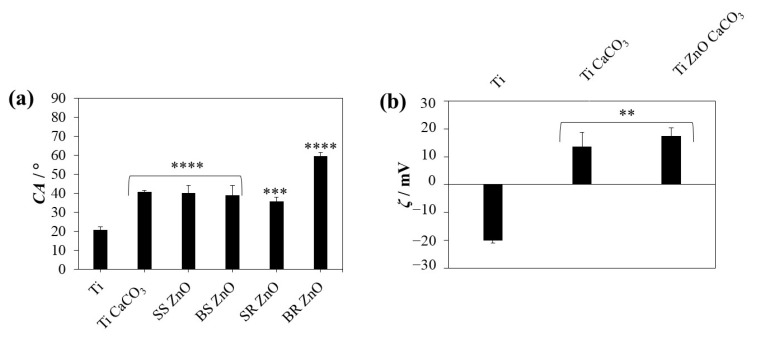
(**a**) Water contact angle (CA) and (**b**) zeta potential (ζ) at pH = 6 of uncoated titanium, titanium with epitaxially overgrown calcium carbonate, and titanium coated with PAH/ALG multilayers containing four types of ZnO nanoparticles and a finishing layer of epitaxially overgrown calcium carbonate. The statistical significance was determined using a one-way ANOVA Tukey’s test compared to uncoated titanium with ** *p* < 0.0073, *** *p* = 0.0001, and **** *p* < 0.0001. SS ZnO and SR ZnO—smaller sized sphere- and rod-like ZnO nanoparticles, respectively, BS ZnO and BR ZnO—larger sized sphere- and rod-like ZnO nanoparticles, respectively.

The surface properties of titanium plates play a crucial role in potential antimicrobial and biomedical applications. For this reason, surface contact angle and surface charge properties were assessed for uncoated titanium, as well as CaCO_3_-overgrown titanium with and without ZnO NPs (Figure 5).

Contact angle measurements revealed that uncoated titanium surfaces exhibited hydrophilic properties, with a contact angle of (20.7 ± 1.6)°. After the application of polyelectrolyte layers and CaCO_3_/ZnO NP coatings, the contact angle increased, with the highest values observed on BR ZnO NP-coated surfaces (59.5 ± 2.1)°, *p* < 0.0001. No significant difference was noted between CaCO_3_-overgrown surfaces with or without SS ZnO, BS ZnO, and SR ZnO NPs. The difference in the contact angles of different ZnO NP morphologies could not be detected due to the low amounts of ZnO present (Figure 3). The surface wettability of the coated titanium plates was influenced by the surface coverage with CaCO_3_ crystals, as well as by the PAH/ALG multilayers with incorporated ZnO NPs. The polyelectrolyte layers modified the surface charge and chemistry, influencing the wettability of the titanium coatings. The positively charged terminating PAH layers increased surface hydrophobicity. Our previous research showed that alginate layers demonstrated slightly lower contact angles (≈45°) compared to PAH layers (≈65°), while nanoparticles of ZnO with various morphologies also decreased the contact angle values (≈40°) [22]. The importance of surface coverage with CaCO_3_ crystals is related to surface smoothness, where smoother surfaces show better wettability [84] than the rougher ones in which PAH terminating layers are trapped in the uncovered areas of the titanium plates. The more pronounced deviation in the contact angle of samples treated with BR ZnO NPs is likely attributable to the significantly lower surface coverage with CaCO_3_ (Figure 4). Due to poor coverage, the PAH molecules used for ZnO immobilisation contributed to a notable increase in the contact angle.

The hydrophilicity of the ZnO-coated surfaces is particularly relevant to protein adsorption, cell adhesion, and bacterial attachment. More hydrophilic surfaces (lower contact angles) tend to promote favourable interactions with osteoblast cells, supporting biocompatibility and osseointegration [85]. The surface contact angles of *S. aureus* strains range from 18° to 25°, *S. epidermidis* from 18° to 33° [86], and *C. albicans* from 29° to 48° [87]. Several studies have demonstrated that microbes with hydrophilic surface properties adhere more easily to hydrophilic surfaces [88,89,90,91].

Zeta potential measurements further confirmed significant differences between the samples (*p* < 0.05). The uncoated titanium surface exhibited a negative surface charge of (−20.1 ± 0.9) mV. However, due to the application of positively charged PAH multilayers, the treated samples demonstrated a positive surface charge, with no significant differences among the ZnO NP-coated samples (*ζ* > 13 mV). Negatively charged bacteria, such as *S. aureus* [92], are expected to adhere less to negatively charged surfaces, due to electrostatic repulsion between negatively charged microbial cells and surfaces [89].

PAH/ALG polyelectrolyte layers were employed for NP encapsulation to minimise direct contact with human cells, inhibit NP release from titanium surfaces, and facilitate Zn^2+^ ion release for antimicrobial activity. The ICP-MS analysis indicated that the percentage of free ZnO NPs released after 24 h of incubation in water was lower for smaller-sized NPs (<11.3%), suggesting stronger NP attachment to the titanium surface compared to BS ZnO NPs (<14%) and BR ZnO NPs (<35%). This effect was primarily attributed to the smaller NP size but also to improved CaCO_3_ surface coverage, which prevented NP release. The significantly lower surface coverage in BR ZnO NP samples led to a markedly higher release of NPs from the surface.

**Figure 6:**
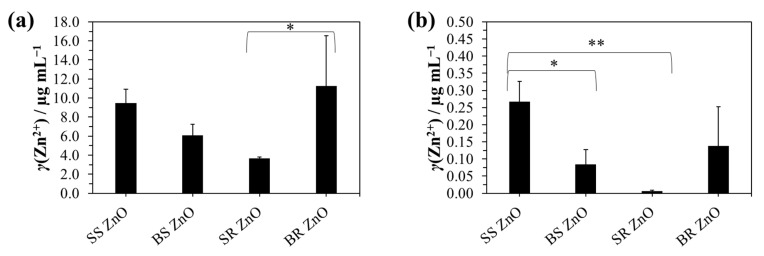
Inductively coupled plasma mass spectrometry analysis of Zn^2+^ ions from the titanium coated with PAH/ALG multilayers containing four types of ZnO nanoparticles and a finishing layer of epitaxially overgrown calcium carbonate: (**a**) after complete ZnO nanoparticle dissolution in HCl and (**b**) after 24 h incubation in water for monitoring the ion release. The statistical significance was determined using a one-way ANOVA Tukey’s test between four types of ZnO nanoparticles * *p* < 0.0426 and ** *p* = 0.0062.

CaCO_3_ overgrowth not only immobilises NPs but may also enable prolonged antimicrobial action due to the release of Zn^2+^ ions as CaCO_3_ gradually resorbs into the bone. The total Zn^2+^ concentration on the titanium surface is presented in Figure 6a, with ZnO NP amounts remaining below 18 µg mL^−1^. The quantified ZnO NP levels on the surface were (4.5 ± 0.2) µg cm^−2^, (7.6 ± 1.5) µg cm^−2^, (11.8 ± 1.8) µg cm^−2^, and (14.0 ± 6.6) µg cm^−2^ for SR ZnO, BS ZnO, SS ZnO, and BR ZnO NPs, respectively.

Zn^2+^ ion release into the aqueous solution was also assessed (Figure 6b), revealing concentrations below 0.4 µg mL^−1^, with significantly lower release observed for BS ZnO NPs (*p* < 0.0426) and SR ZnO NPs (*p* = 0.0062). Hexagonal wurtzite ZnO is a polar crystal with preferential growth along the (0001) direction, where bonding is characterised by a polar covalent nature due to the electronegativity difference between Zn (1.65) and O (3.44), resulting in bond polarisation toward oxygen. This polarity, along with variations in atomic surface structure and surface area, significantly influences Zn^2+^ ion release. Studies [93,94,95] indicate that spherical ZnO nanoparticles are predominantly terminated by polar {0001} facets, with the number and ratio of oxygen and hydroxide groups playing a key role in Zn ion dissolution. Smaller ZnO nanoparticles, such as SS ZnO, have a greater exposed surface area, which facilitates increased Zn^2+^ release [13,20]. SS ZnO exhibited the highest Zn^2+^ release percentage (2.9 ± 1.1%), while SR ZnO, despite its small size, showed the lowest release (0.2 ± 0.1%), highlighting an additional morphology-dependent effect. Spherical ZnO particles (SS ZnO, BS ZnO) generally showed higher Zn^2+^ release percentages compared to rod-shaped particles (SR ZnO, BR ZnO), a trend also observed by Misra et al., where spherical CuO NPs were more soluble than rod- and spindle-shaped NPs [96]. SS ZnO (2.9%) exhibited almost three times the Zn^2+^ release percentage of BS ZnO (1.3%), despite their comparable total ZnO loading, suggesting that smaller spherical particles dissolved more rapidly. In contrast, rod-like ZnO nanoparticles (SR ZnO, BR ZnO) released significantly lower Zn^2+^ percentages (0.2% and 0.9%, respectively), likely due to their anisotropic structure limiting surface dissolution. While the total ZnO content on the multilayers affected Zn^2+^ ion release, the percentage of released Zn^2+^ did not scale proportionally with the total ZnO mass. BR ZnO had the highest total ZnO content (11.24 ± 5.29 µg/mL) but released a lower Zn^2+^ percentage (0.9 ± 0.6%), indicating slower dissolution kinetics despite high loading. Conversely, SS ZnO, with a lower total ZnO content (9.48 ± 1.43 µg/mL), exhibited the highest Zn^2+^ release (2.9%), reinforcing the importance of size-dependent dissolution kinetics over total ZnO mass. SR ZnO had the lowest Zn^2+^ release percentage (0.2%), despite its small size, suggesting that rod-like morphology plays a critical role in limiting ion dissolution. Additionally, ZnO nanoparticles synthesised in alcoholic solvents (ethanol, 1-pentanol) or in the presence of monoethanolamine (SS and SR ZnO) exhibited a larger surface area compared to those prepared in aqueous NaOH solutions (BS and BR ZnO). This difference resulted from the higher proportion of chemisorbed hydroxyl groups and organic residues from precursor salts or solvents, emphasising the structural role of ZnO nanoparticles in determining their antimicrobial performance. Zn^2+^ ions induce microbial cell death by damaging biomolecules through intracellular reactive oxygen species (ROS) generation [97,98,99]. Additionally, Zn^2+^ ions cause increased membrane permeability leading to higher microbial susceptibility to the action of NPs [31].

### 3.3. Antimicrobial Testing of ZnO/CaCO_3_-Coated Titanium Surfaces

**Figure 7:**
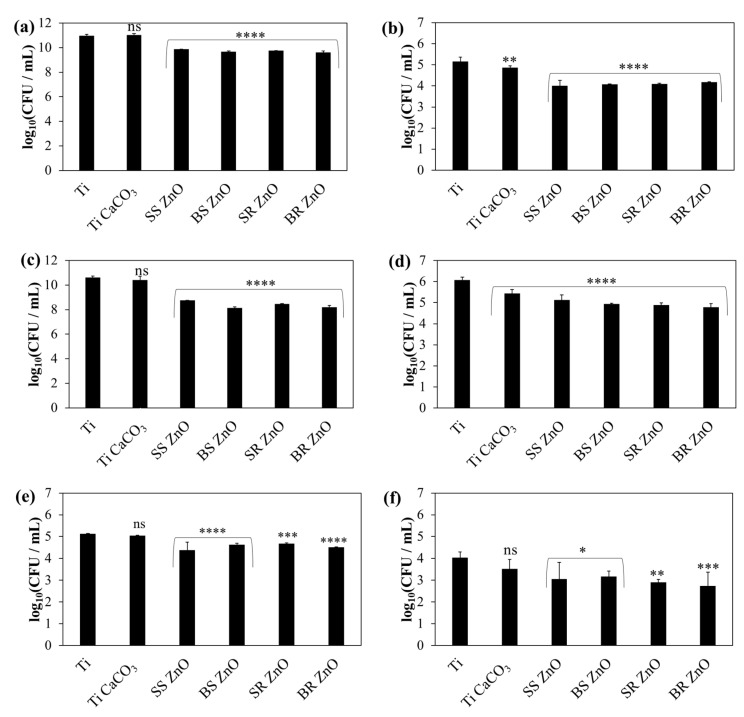
Average bacteria and fungal count per millilitre (CFU/mL) on titanium coated with sphere and rod ZnO NPs with different sizes against *S. aureus* (**a**,**b**), *S. epidermidis* (**c**,**d**), and *C. albicans* (**e**,**f**). The number of viable microbial cells (**a**,**c**,**e**) planktonic and (**b**,**d**,**f**) adhered are shown. The statistical significance was determined using a one-way ANOVA Tukey’s test of the log reduction data points compared to uncoated titanium with * *p* ≤ 0.0346, ** *p* ≤ 0.0095, *** *p* = 0.0005, **** *p* < 0.0001, and ns—nonsignificant.

The antimicrobial tests were conducted with the *S. aureus* ATCC 25923, *S. epidermidis* ATCC 14990, and *C. albicans* ATCC 36232 strains. A one-way analysis of variance (ANOVA) was performed to assess the statistical differences in microbial viability based on ZnO NP morphology. Figure 7 depicts the number of viable planktonic and adhered *S. aureus*, *S. epidermidis,* and *C. albicans* on titanium plates coated with PAH/ALG polyelectrolyte multilayers and CaCO_3_ crystals without and with ZnO NPs exhibiting different morphological features.

For planktonic *S. aureus* cells (Figure 7a), only the ZnO NP-coated plates exhibited a significant reduction in cell viability (*p* < 0.0001), achieving a viability reduction of over 92% (Table S5). Among the different ZnO NP morphologies, SS ZnO NP-coated surfaces caused a greater decrease in cell viability than BS ZnO NP (*p* = 0.0145) and BR ZnO NP (*p* = 0.0003).

For adhered *S. aureus* cells (Figure 7b), titanium plates coated with pure CaCO_3_ demonstrated a slight reduction in viability (*p* = 0.0095). However, ZnO NP-coated surfaces, regardless of morphology, led to a substantial decrease in viability (*p* < 0.0001, *p* > 92%) (Table S5), with no statistically significant differences between ZnO NP morphologies (*p* > 0.9163).

Planktonic *S. epidermidis* cells (Figure 7c, Table S5) exhibited a significant reduction in viability (*p* < 0.0001) only on ZnO NP-coated surfaces, with reductions exceeding 98%. Comparing ZnO NP morphologies, SR ZnO NP-coated surfaces demonstrated a greater decrease in cell viability than SS ZnO NPs (*p* = 0.0293), BS ZnO NPs (*p* = 0.0128), and BR ZnO NPs (*p* = 0.0495).

For adhered *S. epidermidis* cells (Figure 7d, Table S5), the viability significantly decreased on titanium plates treated with CaCO_3_ and PAH/ALG polyelectrolyte multilayers containing ZnO NPs (*p* < 0.0001, *p* > 88%). BR ZnO-coated surfaces exhibited the highest reduction in cell viability (*p* = 0.0131).

For planktonic *C. albicans* cells, titanium plates coated with pure CaCO_3_ did not reduce fungal viability (*p* = 0.9031). However, ZnO NP-coated surfaces significantly decreased fungal viability (*p* < 0.0001), with the highest reduction reaching 79% (Table S5). A statistically significant difference was observed between SS ZnO NPs and SR ZnO NPs (*p* = 0.0262), where SS ZnO NPs exhibited a weaker antifungal effect. No significant differences were detected among the other ZnO morphologies (*p* > 0.0953).

For adhered *C. albicans* cells, titanium plates coated with pure CaCO_3_ had no impact on viability (*p* > 0.05). However, surfaces coated with both CaCO_3_ and ZnO NPs significantly reduced viability (*p* < 0.0346) (Figure 7f, Table S5). Surfaces coated with SS ZnO NPs and BS ZnO NPs (*p* < 0.0346) reduced viability by over 87%, while SR ZnO NPs (*p* = 0.0030) and BR ZnO NPs (*p* = 0.0005) achieved the highest reduction (*p* > 94%).

For adhered *S. aureus* and *S. epidermidis* cells, slight antimicrobial activity was observed on CaCO_3_-overgrown samples without ZnO NPs, likely due to the action of PAH molecules. However, this effect was less pronounced compared to ZnO NPs. Previous studies have demonstrated the antimicrobial properties of PAH against *S. aureus* [21,100,101]. In this study, PAH molecules affected only adhered cells, whereas planktonic cell viability remained unchanged, indicating that direct contact with the PAH-treated surface was necessary.

In contrast, ZnO NP-coated samples released Zn^2+^ ions, which inactivated both adhered and planktonic cells. When comparing the effectiveness of ZnO NPs against planktonic and adhered microbial cells, distinct trends were observed. The antimicrobial tests revealed that for *S. aureus*, SS ZnO NPs exhibited the highest reduction in planktonic cell viability, significantly outperforming BS ZnO NPs and BR ZnO NPs. Smaller ZnO nanoparticles have a higher surface area-to-volume ratio [13,20], increasing their interaction with bacterial cells and enhancing Zn^2+^ ion release, leading to greater microbial inactivation. A similar trend was observed for *S. epidermidis* and *C. albicans*, where SR ZnO NPs displayed the strongest antimicrobial effect. When comparing various NP morphologies (flowers, rods, plates, spheres) studied for metal NPs (Ag, ZnO, etc.), the overall conclusion suggests that NP size, rather than morphology, is the primary determinant of antibacterial activity, due to the faster dissolution and stronger effect of smaller particles [102,103,104,105]. The difference in antimicrobial performance between spherical (SS ZnO, BS ZnO) and rod-like (SR ZnO, BR ZnO) morphologies is evident in Figure 7. Rod-like ZnO nanoparticles (SR ZnO, BR ZnO), particularly at smaller sizes, were more effective at damaging bacterial membranes, possibly due to increased surface contact area and mechanical puncturing effects. BR ZnO (large rod-like morphology), despite slower dissolution rates, still exhibited significant antimicrobial activity in adhered cells, likely due to its direct contact effects on microbes rather than ion release alone. Furthermore, PAH has intrinsic antimicrobial properties due to its positively charged nature, which can further disrupt bacterial membranes via electrostatic interactions [21,100,101]. Among the prepared NPs, SR ZnO demonstrated the slowest Zn^2+^ release (0.2%), despite its small size, suggesting that its rod-like morphology played a role in its antibacterial activity against *S. epidermidis* and *C. albicans*. The observed activity most likely stemmed from the direct interaction of released SR ZnO particles from the multilayers, which damaged the cell membrane due to NP adhesion to microbial cells and penetration through the membrane [106]. The stress stimuli may also be influenced by particle shape, as some research has shown that rod-like NPs exhibit enhanced antibacterial activity compared to similarly sized flower-like ZnO NPs [107]. For planktonic cells, the antifungal activity of ZnO NPs was lower compared to bacterial strains, with a maximum viability reduction of 79%. One possible explanation for these differences is the distinct physicochemical properties of each morphology and their interactions with microbial cells. The combination of small ZnO nanoparticle size, high Zn^2+^ ion release, and rod-like morphology optimises antimicrobial effects. Smaller particles (SS ZnO, SR ZnO) release Zn^2+^ more effectively and interact more directly with bacterial cells, while rod-like ZnO (SR ZnO, BR ZnO) enhances membrane penetration and mechanical disruption. The synergistic effects of Zn^2+^ ion release and NP morphology make ZnO-coated surfaces highly effective for antimicrobial applications, with the potential for infection-resistant biomedical coatings.

Notably, adhered *S. aureus* cells exhibited a similar level of viability reduction across all ZnO-coated surfaces, suggesting that direct contact with the immobilised ZnO NPs played a crucial role in *S. aureus* inactivation. When analysing adhered *S. epidermidis* and *C. albicans* cells, the highest viability reduction was observed with BR ZnO. The BR ZnO caused the highest viability reduction on contact because of the poor surface coverage with CaCO_3_ crystals.

The difference in the antimicrobial activity of ZnO NPs was further analysed based on the percentage reduction in cell viability for all three strains (Table S5). A two-way ANOVA confirmed significant differences in planktonic cell viability reduction among *S. aureus*, *S. epidermidis*, and *C. albicans* (*p* < 0.0012). Notably, for all ZnO NPs, the viability reduction was significantly lower for *C. albicans* compared to the bacterial strains (*p* < 0.0459). Between the bacterial strains, *S. epidermidis* exhibited a greater viability reduction than *S. aureus* (*p* < 0.0211) on ZnO NP-coated titanium surfaces. Previously it was observed that the minimal inhibitory concentration of ZnO particles was much higher for fungi *Aspergillus flavus*, *Aspergillus nidulans*, *Trichoderma harzianum*, and *Rhizopus stolonifer* than for bacteria *S. aureus*, *Serratia marcescens*, *Proteus mirabilis*, and *Citrobacter freundii* [108]. Furthermore, higher ZnO NPs antimicrobial activity with different ZnO NPs was observed for *Escherichia coli*, *Pseudomonas aeruginosa*, and *S. aureus* than for *C. albicans* and *Aspergillus brasiliensis* fungi, with NPs with smaller sizes causing higher antimicrobial effect [109]. Fungi exhibit stronger resistance to metal NPs than bacteria due to their thicker cell walls, superior detoxification mechanisms, metal ion tolerance, biofilm formation, and slower metabolic activity [110,111].

For adhered cells, viability reduction differences among the three microbial strains were not statistically significant (*p* = 0.9042). Overall, the results demonstrated that direct contact with CaCO_3_ and ZnO NP-treated surfaces effectively eliminated all three microbial strains. However, in planktonic cell contamination—where Zn^2+^ concentrations are presumably lower—bacteria displayed higher sensitivity than *C. albicans*.

The antimicrobial activity of ZnO NPs is attributed to multiple mechanisms, including Zn^2+^ ion release, the slight production of H_2_O_2_, and the presence of oxygen vacancies on the ZnO surface [112,113]. Small amounts of H_2_O_2_ can form even in dark conditions via the conversion of H_2_O into H_2_O_2_, facilitated by ZnO surface oxygen vacancies [112]. The combination of Zn^2+^ ions and generated H_2_O_2_ significantly enhances antimicrobial effects [114]. Zn^2+^ ions contribute to microbial cell death through the generation of reactive oxygen species, protein mis-metallation, and cellular dysfunction. These processes can induce lipid peroxidation, nucleic acid damage, and protein oxidation [97,98]. Furthermore, Zn^2+^ ions increase membrane permeability, making cells more susceptible to the NP action [31].

For planktonic cells, direct interaction with ZnO NPs is possible, as ICP-MS measurements confirmed that some NPs detached from the titanium surface into the solution. Adhered microbes, in contrast, interact directly with immobilised NPs, which may disrupt cellular energy metabolism by increasing sugar metabolism and pyrimidine biosynthesis while decreasing amino acid synthesis [97].

### 3.4. The Result of In Vitro Cytocompatibility Test

Angiogenesis and osteogenesis play crucial roles in bone healing and skeletal development. Both polyelectrolytes used in this study (PAH, ALG) have previously demonstrated good biocompatibility with human cells [115,116,117,118,119,120]. The in vitro cytocompatibility of Zn^2+^ ions and ZnO NPs (SS, BS, SR, BR) was assessed using the osteoblast precursor cell line MC3T3-E1. An indirect viability assay was conducted under varying Zn^2+^ concentrations (0.5–20) µg mL^−1^ and ZnO NPs (0–50) µg mL^−1^ to evaluate their potential toxicity.

**Figure 8:**
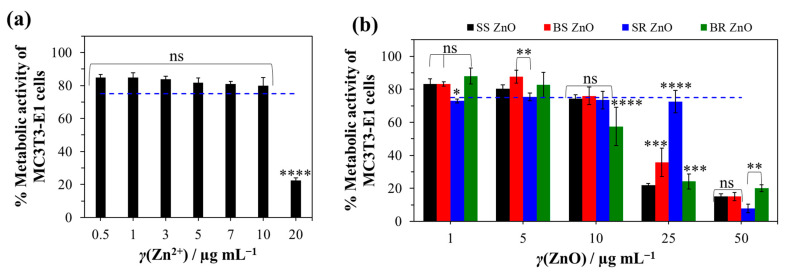
Metabolic activity of MC3T3-E1 cells under different Zn^2+^ (**a**) and ZnO nanoparticle (**b**) concentrations. The decrease in the metabolic activity of the cells was determined in relation to the medium without Zn^2+^ ions and ZnO nanoparticles. The statistical significance was determined using one-way (**a**) and two-way (**b**) ANOVA tests with * *p* < 0.0231, ** *p* < 0.0093, *** *p* < 0.0005, **** *p* ≤ 0.0001, and ns—nonsignificant. The blue dashed line indicates the cut-off viability between non-toxic and toxic properties according to ISO 10993-5 [125].

Changes in MC3T3-E1 metabolic activity (Figure 8a) indicated a significant reduction in cell viability, with the most pronounced cytotoxic effect observed at the highest Zn^2+^ concentration (*p* < 0.0001), where viability decreased to (22 ± 2)%. The ICP-MS analysis (Figure 6b) indicated the highest release of Zn^2+^ ions was below 0.35 µg mL^−1^, with a total potential release after complete ZnO NP dissolution (Figure 6a) being (11.2 ± 5.3) µg mL^−1^.

The biocompatibility assessment demonstrated a cytotoxic effect only at the highest tested Zn^2+^ ion concentration, (20 µg mL^−1^, *p* < 0.0001). Notably, the released number of Zn^2+^ ions from the coated titanium surface (Figure 6b) remained significantly lower than the concentration (20 µg mL^−1^) associated with MC3T3-E1 cell cytotoxicity (Figure 8). However, the total Zn^2+^ concentration (*γ*(Zn^2+^) = (11.2 ± 5.3) µg mL^−1^) was near the limit associated with cytotoxic effects. Therefore, further ZnO NP treatment should ensure Zn^2+^ ion concentrations do not exceed 10 µg mL^−1^. This could be achieved through the precise control of ZnO NP deposition using a spin-coating technique, allowing for optimal NP distribution beneath the CaCO_3_ layer.

Zn^2+^ ions are crucial in cellular pathways; they facilitate normal cell functions and are beneficial to cells in low concentrations [31]. Studies on biodegradable zinc alloys have demonstrated enhanced cytocompatibility, osseointegration, and osteogenesis, highlighting the therapeutic potential of Zn^2+^ ions [121]. Additionally, ZnO-incorporated chitosan scaffolds (3 wt%) have been shown to promote MC3T3-E1 cell proliferation, adhesion, and differentiation [122], reinforcing the potential of ZnO for implant development. This has been further confirmed by research into ZnO-based magnesium orthopaedic implants [123] and polycaprolactone/hydroxyapatite/ZnO films [124].

The viability of cells decreased significantly depending on the concentration and morphology of NPs (Figure 8b). Considering the limit value of viability (75%) according to ISO 10993-5 [125], the concentration for SS, BS, and BR ZnO was 10 μg mL^−1^, and that for SR ZnO was 1 μg mL^−1^. As expected, the size of the NPs played a decisive role—smaller NPs showed higher toxicity compared to the large BS and BR ZnO, which is consistent with the literature [126,127,128]. This size-dependent cytotoxicity is attributed to enhanced cellular uptake and increased surface reactivity. Therefore, when designing NPs for biomedical applications, it is essential to carefully consider their size to balance efficacy and safety.

Additionally, increasing the concentration of NPs might also lead to higher cytotoxicity. The most significant reduction in cell viability (<40%) was observed for SS, BS, and BR ZnO NPs at 25 µg/mL (*p* < 0.0005) and for SR ZnO NPs at 50 µg/mL (*p* < 0.0093). Among the different NPs tested, SR ZnO exhibited the highest cytotoxicity (*p* < 0.0132). Interestingly, a similar trend was observed in antimicrobial activity, where SR ZnO demonstrated the strongest effects against S. epidermidis and C. albicans (Figure 7).

Besides size and concentration, the shape of NPs also influences cytotoxic properties [129,130,131,132]. Non-spherical NPs (e.g., rods, stars, needles, and plate-like structures) often exhibit higher cytotoxicity than spherical ones due to their larger aspect ratio and mechanical interactions with cells [133]. While spherical NPs are internalized more efficiently via endocytosis, rod-like and needle-like NPs can physically damage cell membranes, increasing their cytotoxic potential [133]. Rod-shaped NPs can puncture cell walls, inducing oxidative stress and inflammation [134]. The combination of a high aspect ratio and increased surface energy contributes to their elevated cytotoxicity, highlighting NP shape as a critical factor in designing safe nanomaterials.

Considering both antimicrobial efficacy (Figure 7) and cytotoxicity (Figure 8b), SS, BS, and BR ZnO NPs appear to be better candidates for biomedical applications due to their safer profile while maintaining antibacterial activity.

Previous studies have indicated that loosely bound ZnO NPs on orthotic materials can induce cytotoxic effects in human epithelial cells [135]. To mitigate this risk, the application of polyelectrolyte multilayers and CaCO_3_ above ZnO NPs was employed to immobilise the NPs, allowing for controlled Zn^2+^ ion release and prolonged bioactivity. While this study provides initial insights into coating stability during short-term biological interactions, future work should explore long-term degradation, mechanical resilience, and coating durability under conditions mimicking physiological environments (pH-dependent stability tests, high-ionic-strength solutions, and in vivo studies). Since CaCO_3_ is gradually resorbed by bone cells, this coating strategy enhances the long-term functionality of the implant.

However, due to weaker surface coverage with CaCO_3_ crystals (Figure S9b–e), further research is required to improve crystal precipitation. One possible strategy is to coat CaCO_3_ surfaces with ZnO NPs, as uncoated surfaces have shown excellent coverage with a high density of vaterite crystals (Figure 4a and Figure S8a). However, such an approach could heighten the cytotoxic effects of ZnO NPs due to direct contact with human cells [30]. Conversely, covering ZnO NPs with CaCO_3_ facilitates their gradual dissolution, ensuring a prolonged antimicrobial effect.

ZnO NPs and CaCO_3_ coatings may provide long-term antimicrobial efficacy, particularly in cases of local infection. A decrease in pH caused by infection could accelerate the dissolution of CaCO_3_ and ZnO NPs, leading to sustained antimicrobial activity even after implantation. However, further studies are needed to evaluate the influence of CaCO_3_ coatings and the optimal ZnO NP deposition method—whether beneath, within, or on top of the CaCO_3_ layer—to develop an ideal composite material.

Additionally, implants coated with vaterite, an unstable and highly soluble CaCO_3_ polymorph, in combination with ZnO NPs should be investigated in vivo. Given the promising antimicrobial properties observed in vitro, further research is necessary to determine the most effective application method that ensures safety and promotes rapid recovery following titanium bone implant placement.

## 4. Conclusions

This study successfully developed a novel antimicrobial and biocompatible titanium surface coating, incorporating ZnO NPs with diverse morphologies, immobilised within PAH and alginate multilayers, alongside epitaxially grown calcium carbonate (CaCO_3_) in the form of vaterite crystals. The antimicrobial properties of the coating were evaluated against Gram-positive bacteria (*Staphylococcus aureus* and *Staphylococcus epidermidis*) and the yeast *Candida albicans*, all of which are common pathogens associated with implant-related infections. The key findings are summarised below:

- A stable CaCO_3_ coating featuring epitaxially grown vaterite crystals was successfully prepared in a system containing 0.02 mol dm^−3^ equimolar Ca^2+^/HCO_3_^2−^, 5 ppm pAsp, and 0.1 mol dm^−3^ NaCl.
- The presence of ZnO NPs induced a new form of flower-like vaterite morphology and influenced vaterite crystal growth.
- Titanium surfaces treated with this coating exhibited robust antimicrobial activity, achieving over 90% reduction in microbial viability for *S. aureus*, *S. epidermidis*, and *C. albicans*. In direct contact with the treated surface, all three microbial strains were equally eradicated. However, in contamination further from the surface (planktonic cells), where the Zn^2+^ ion concentration was presumably lower, bacterial cells exhibited much greater sensitivity than *C. albicans* cells. The antimicrobial efficacy of the coating was influenced by the morphology of ZnO NPs, with certain morphologies (e.g., SS ZnO and SR ZnO) demonstrating a stronger reduction in microbial viability, particularly against *S. aureus* and *S. epidermidis*, while others exhibited lower but still significant antimicrobial effects, highlighting the role of the NP structure in antimicrobial performance.
- The concentration of Zn^2+^ ions released from the coating remained below cytotoxicity thresholds for MC3T3-E1 cells, highlighting the biocompatibility and safety of the coating for implant applications. The combination of high aspect ratio (elongated shapes) and high surface energy led to increased cytotoxicity, making the NP shape a critical factor in designing safe nanomaterials. Among the used NPs, SS, BS, and BR ZnO NPs emerged as better candidates for biomedical applications due to their safety and expressed antibacterial activity.

These findings highlight the potential of the CaCO_3_/ZnO NP composite coating as a promising approach for developing antimicrobial and biocompatible titanium implants. Future research should focus on optimising the coating process, assessing long-term stability, and evaluating performance under in vivo conditions. These steps are essential for translating this method into clinical applications, ensuring both safety and enhanced recovery following titanium bone implant procedures.

## Data Availability

The original contributions presented in this study are included in the article and Supplementary Materials. Further inquiries can be directed to the corresponding author.

## Supplementary Materials

The following supporting information can be downloaded at: https://www.mdpi.com/article/10.3390/jfb16030108/s1, Figure S1: PXRD diffraction intensity of ZnO nanoparticles at specific 2*θ* angles; Figure S2: SS ZnO particle morphology; Figure S3: ZnO particle size distribution; Figure S4: SEM images and FTIR spectrum of surface-activated titanium by H_2_O_2_; Figure S5: SEM images with EDS mapping and point spectra showing the atom composition on titanium plates treated with SS ZnO; Figure S6: SEM images with EDS mapping and point spectra showing the atom composition on titanium plates treated with SR ZnO; Figure S7: FTIR and PXRD characterisation of calcium carbonate precipitated in the presence of polyaspartic acid on the glass surface of the laboratory beaker during epitaxial growth experiments; Figure S8: Composition of calcium carbonate precipitated in the presence of polyaspartic acid on the glass surface of the laboratory beaker during epitaxial growth experiments; Figure S9: SEM images of titanium plate surfaces after calcium carbonate epitaxial growth experiments on titanium (a), and titanium coated with PAH/ALG multilayers containing four types of ZnO nanoparticles: SS ZnO (b) and SR ZnO (d)—smaller sized sphere- and rod-like ZnO nanoparticles, respectively, BS ZnO (c) and BR ZnO (e)—larger sized sphere- and rod-like ZnO nanoparticles, respectively, ALG—alginate, PAH—poly(allylamine hydrochloride); Table S1: EDS determined the atomic composition of the sample surface corresponding to Figure S5; Table S2: EDS determined the atomic composition of the sample surface corresponding to Figure S6; Table S3: Assignment of IR bands in FTIR spectra of calcium carbonate on coated titanium plates; Table S4: Preparation conditions for epitaxially grown calcium carbonate; Table S5: Percentage reduction (P) in planktonic and adhered *Staphylococcus aureus*, *Staphylococcus epidermidis*, and *Candida albicans* cell viability on titanium coated with sphere and rod ZnO nanoparticles and calcium carbonate.
