# Supplementary material for: ZnO Nanoparticle-Infused Vaterite Coatings: A Novel Approach for Antimicrobial Titanium Implant Surfaces
Source: J Funct Biomater. 2025 Mar 19;16(3):108. doi: 10.3390/jfb16030108 (PMC11943299; doi:10.3390/jfb16030108)
Supplement: Supplementary file 1 [file jfb-16-00108-s001.zip › jfb-3506389-supplementary.pdf]

ZnO Nanoparticle-Infused Vaterite Coatings: A Novel Approach for Antimicrobial Titanium Implant Surfaces

Atiđa Selmani[1], Scarlett Zeiringer[1], Ankica Šarić[2], Anamarija Stanković[3], Aleksander Učakar[4], Janja Vidmar[4], Anže Abram[4], Branka Njegić Džakula[2], Jasminka Kontrec[2], Anamarija Zore[5], Klemen Bohinc[5], Eva Roblegg[1], Nives Matijaković Mlinarić[2*]

1 Pharmaceutical Technology and Biopharmacy, Institute of Pharmaceutical Sciences, University of Graz, Universitätsplatz 1, 8010 Graz, Austria, A.S. atida.selmani@uni-graz.at; S.Z. scarlett.zeiringer@uni-graz.at; E.R. eva.roblegg@uni-graz.at

2 Ruđer Bošković Institute, Bijenička cesta 54, 10000 Zagreb, Croatia; N.M.M. nmatijak@irb.hr; A.Š. Ankica.Saric@irb.hr; B.Nj.Dž. bnjeg@irb.hr; J.K. Jasminka.Kontrec@irb.hr

3 Department of Chemistry, University of Osijek, Ulica cara Hadrijana 8/A, 31000 Osijek, Croatia, A.S. astankovic@kemija.unios.hr

4 Jožef Stefan Institute, Jamova cesta 39, 1000 Ljubljana, Slovenia; J.V. janja.vidmar@ijs.si; A.A. anze.abram@ijs.si

5 Faculty of Health Sciences, University of Ljubljana, Zdravstvena pot 5, 1000 Ljubljana, Slovenia, K.B. klemen.bohinc@zf.uni-lj.si; A.Z. anamarija.zore@zf.uni-lj.si

* Correspondence: nmatijak@irb.hr

Contents

Figure S1. PXRD diffraction intensity of ZnO nanoparticles at specific 2θ angles for SS ZnO (green line), BS ZnO (red line), SR ZnO (black line), and BR ZnO (blue line). 1

Figure S2. SEM images showing the morphology of SS ZnO at various magnifications. 2

Figure S3. Size distribution was determined from the SEM images of a) SS ZnO (small-sized spheres), b) SR ZnO (small-sized rods), c) BS ZnO (bigger-sized spheres), and d) BR ZnO (bigger-sized rods). 3

Figure S4. a) Representative Fourier transformed infrared spectra showing the sample absorbance (A) at a specific wavenumber (v / cm^{-1}). A spectrum of unmodified titanium and surface-activated titanium by H_2O_2 is shown. B) SEM images of surface-activated titanium by H_2O_2....3

Figure S5. SEM images with EDS mapping and point spectra showing the atom composition on titanium plates treated with SS ZnO. 4

Figure S6. SEM images with EDS mapping and point spectra showing the atom composition on titanium plates treated with SR ZnO. 5

Figure S7. Characterization of calcium carbonate precipitated in the presence of polyaspartic acid on the glass surface of the laboratory beaker during epitaxial growth experiments. (a) Representative Fourier transformed infrared spectra showing the sample absorbance (A) at specific wavenumber (v / cm^{-1}) and (b) X-ray diffraction intensity of calcium carbonate at specific 2θ angles.

The standard diffraction reflections of calcite (blue bars) and vaterite (red bars) are shown for comparison. 6

Figure S8. Composition of calcium carbonate precipitated in the presence of polyaspartic acid on the glass surface of the laboratory beaker during epitaxial growth experiments. 6

Figure S9. SEM images of titanium plate surfaces after calcium carbonate epitaxial growth experiments on titanium (a), and titanium coated with PAH/ALG multilayers containing four types of ZnO nanoparticles: SS ZnO (b) and SR ZnO (c) – smaller sized sphere- and rod-like ZnO nanoparticles respectively, BS ZnO (d) and BR ZnO (e) – larger sized sphere- and rod-like ZnO nanoparticles respectively, ALG – alginate, PAH – poly(allylamine hydrochloride). Red arrows show calcite crystals. 7

Table S1. EDS determined the atomic composition of the sample surface corresponding to Figure S5. 8

Table S2. EDS determined the atomic composition of the sample surface corresponding to Figure S6. 8

Table S3. Assignment of IR bands in FTIR spectra of calcium carbonate on coated titanium plates. 8

Table S4. Preparation conditions for epitaxially grown calcium carbonate on titanium surfaces without ZnO NPs for finding the preparation method determined based on semiquantitative FTIR analysis. 9

Table S5. Percentage reduction (*P*) of planktonic and adhered *Staphylococcus aureus*, *Staphylococcus epidermidis* and *Candida albicans* cell viability on titanium coated with sphere and rod ZnO nanoparticles and calcium carbonate. 9

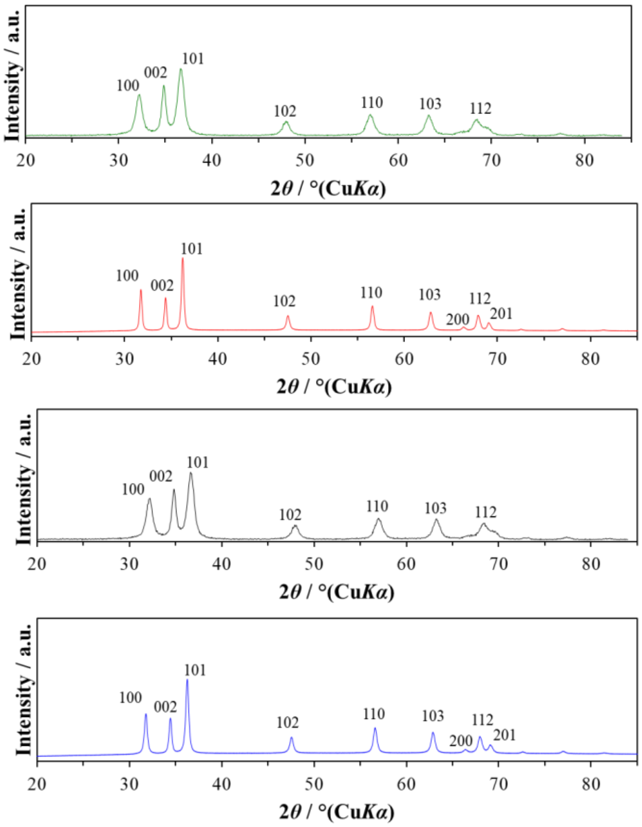

Figure S1. PXRD diffraction intensity of ZnO nanoparticles at specific 2θ angles for SS ZnO (green line), BS ZnO (red line), SR ZnO (black line), and BR ZnO (blue line).

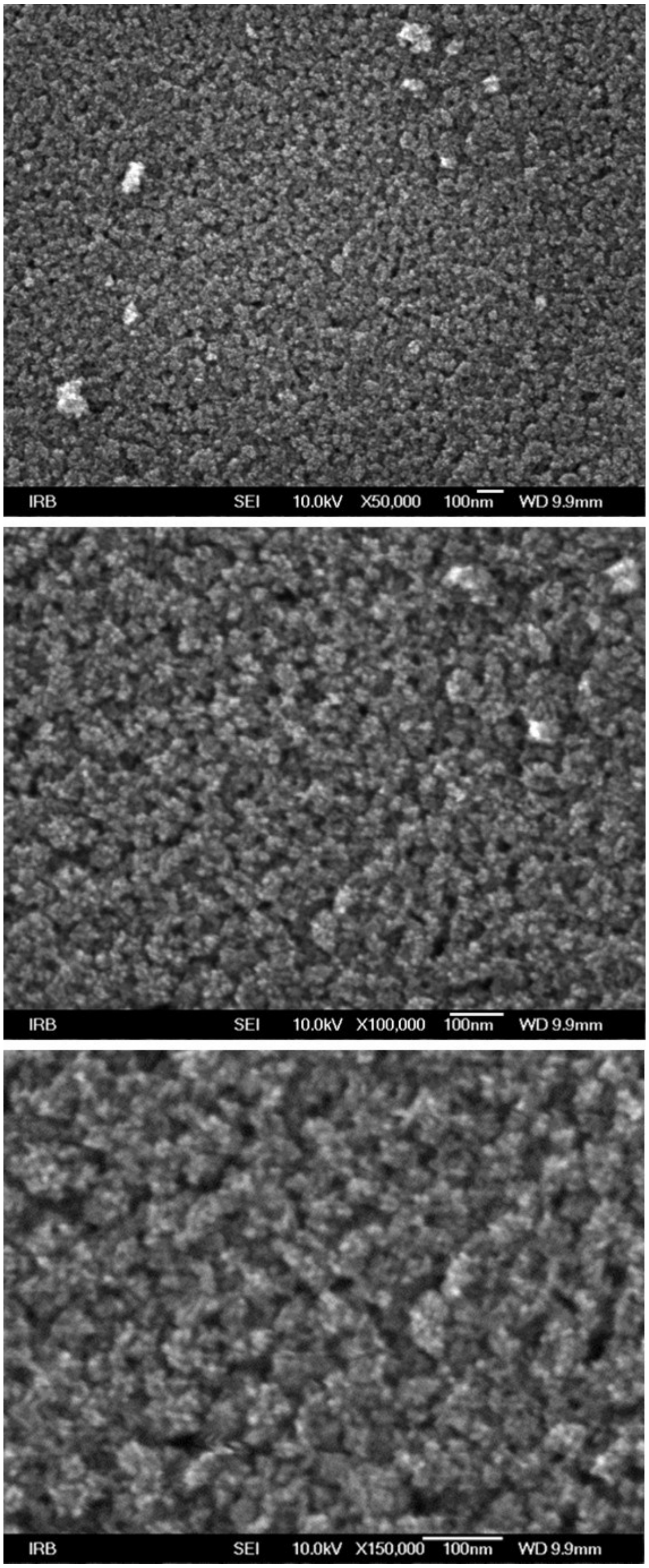

Figure S2. SEM images showing the morphology of SS ZnO at various magnifications.

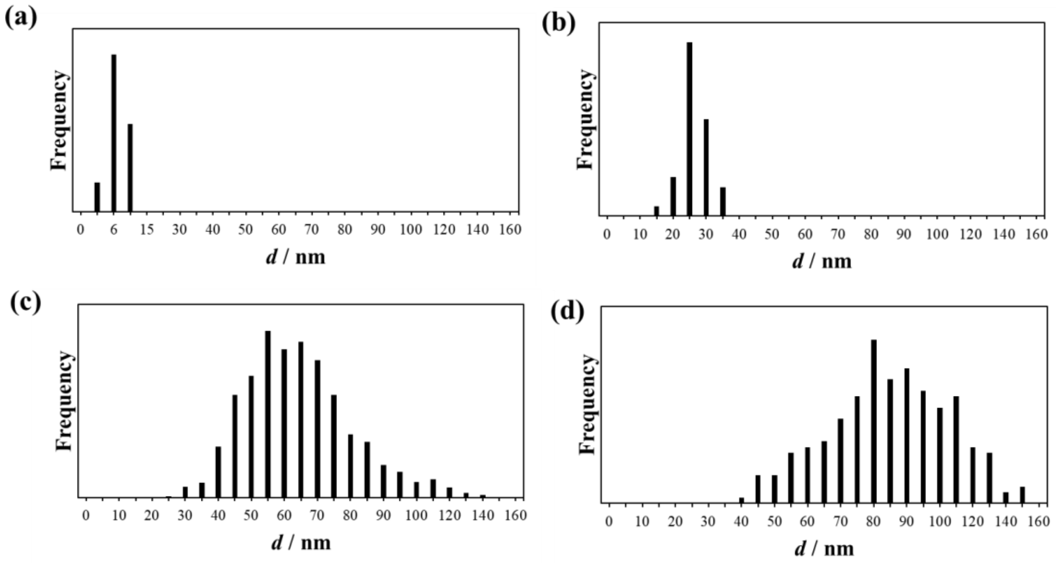

Figure S3. Size distribution was determined from the SEM images of a) SS ZnO (small-sized spheres), b) SR ZnO (small-sized rods), c) BS ZnO (bigger-sized spheres), and d) BR ZnO (bigger-sized rods).

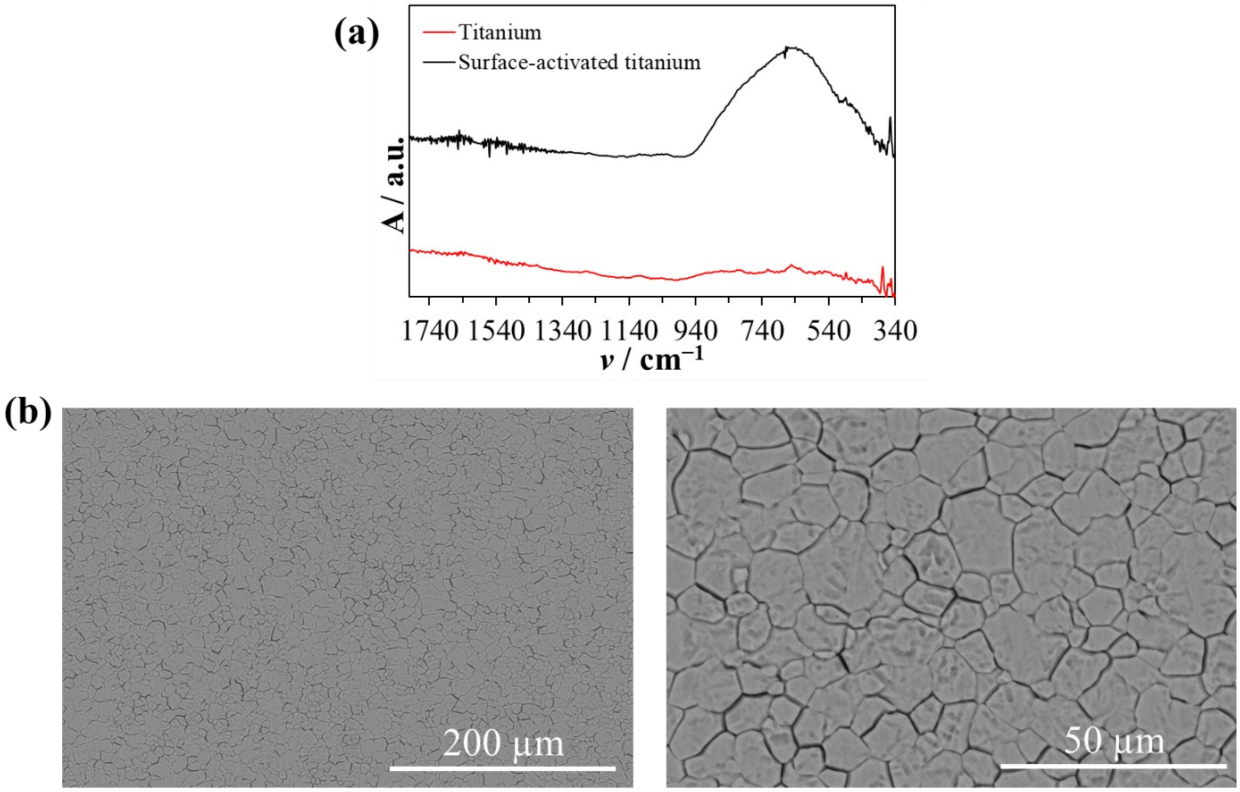

Figure S4. a) Representative Fourier transformed infrared spectra showing the sample absorbance (A) at a specific wavenumber (v / cm^{-1}). A spectrum of unmodified titanium and surface-activated titanium by H_2O_2 is shown. B) SEM images of surface-activated titanium by H_2O_2.

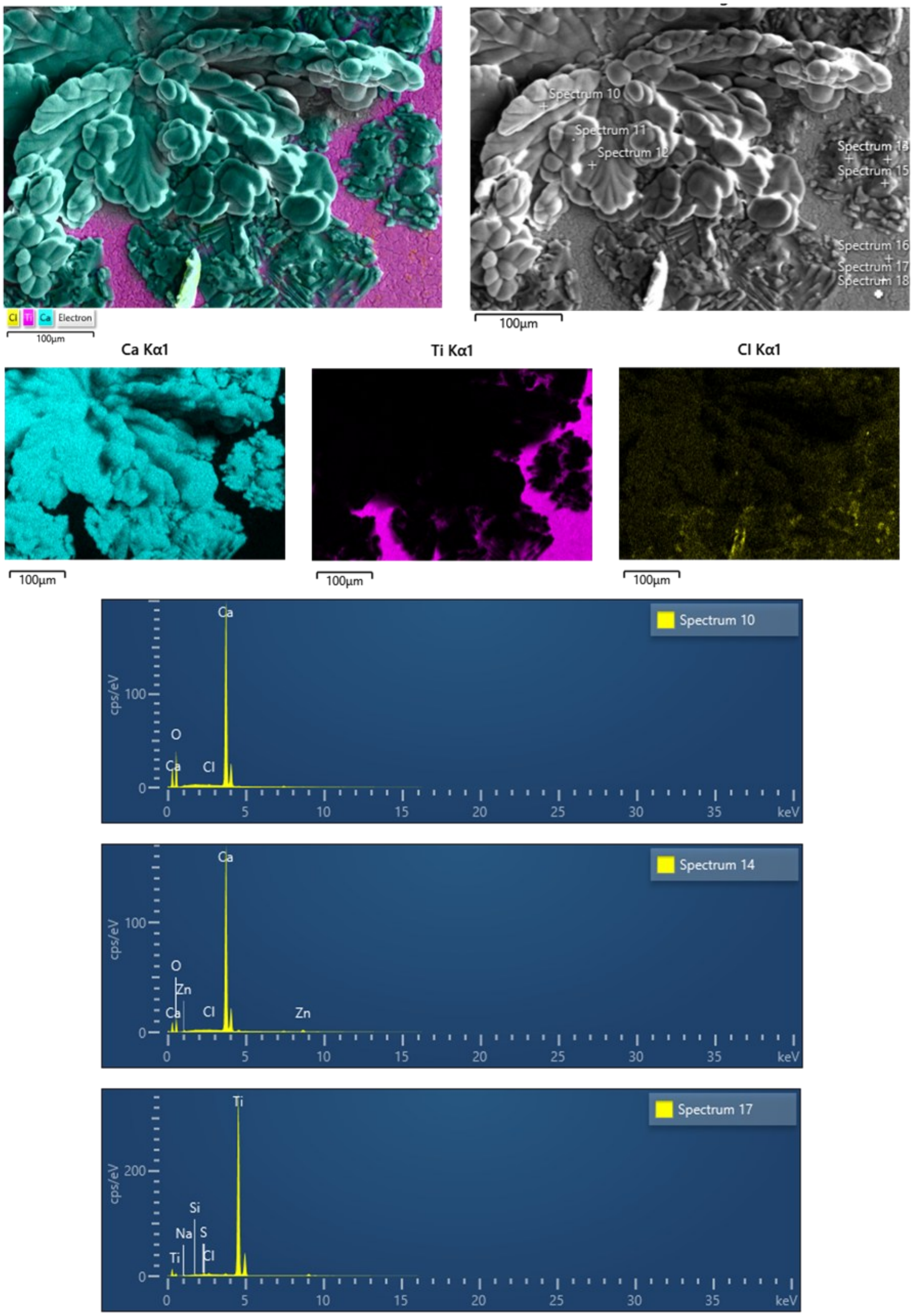

Figure S5. SEM images with EDS mapping and point spectra showing the atom composition on titanium plates treated with SS ZnO.

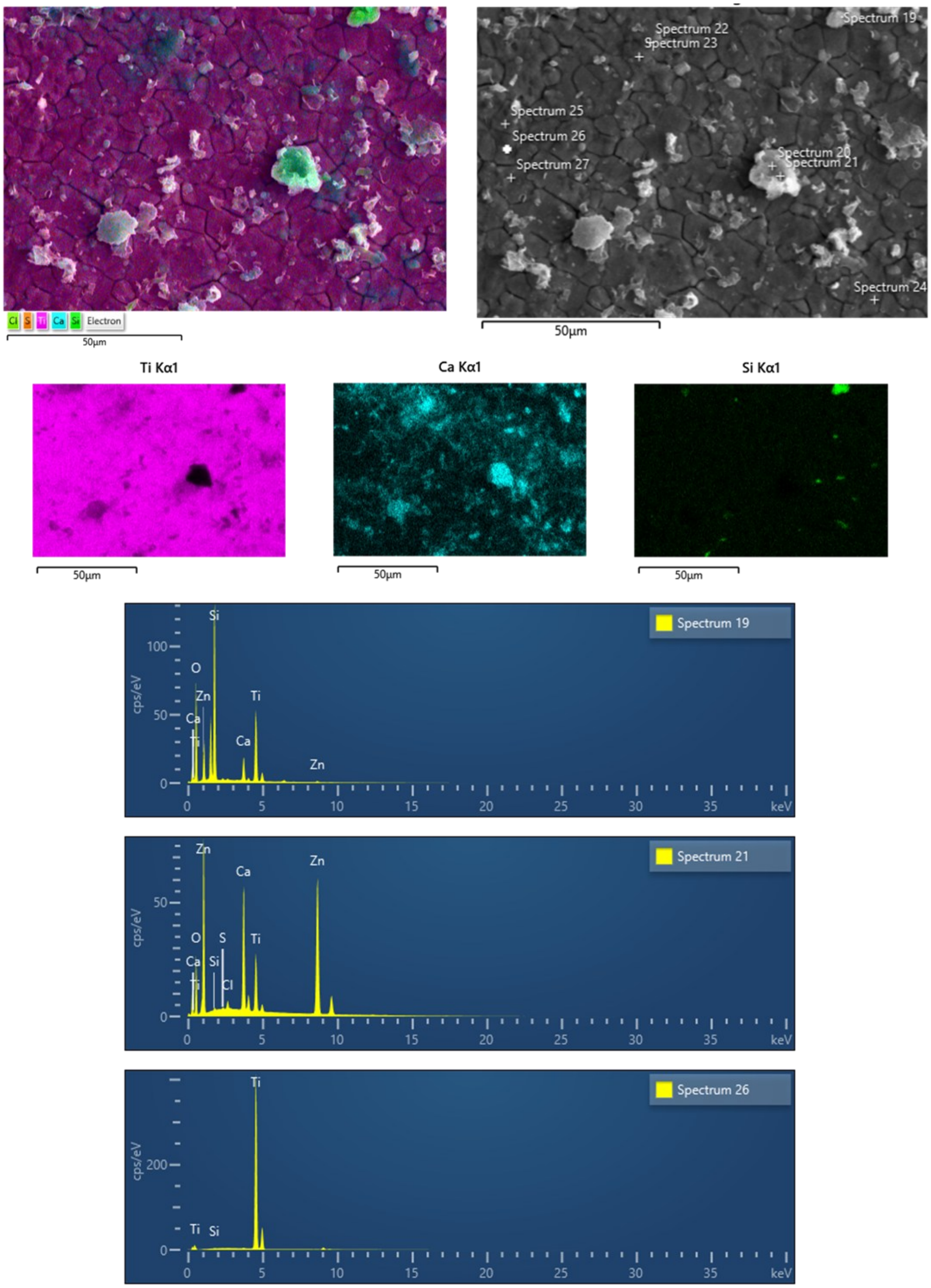

Figure S6. SEM images with EDS mapping and point spectra showing the atom composition on titanium plates treated with SR ZnO.

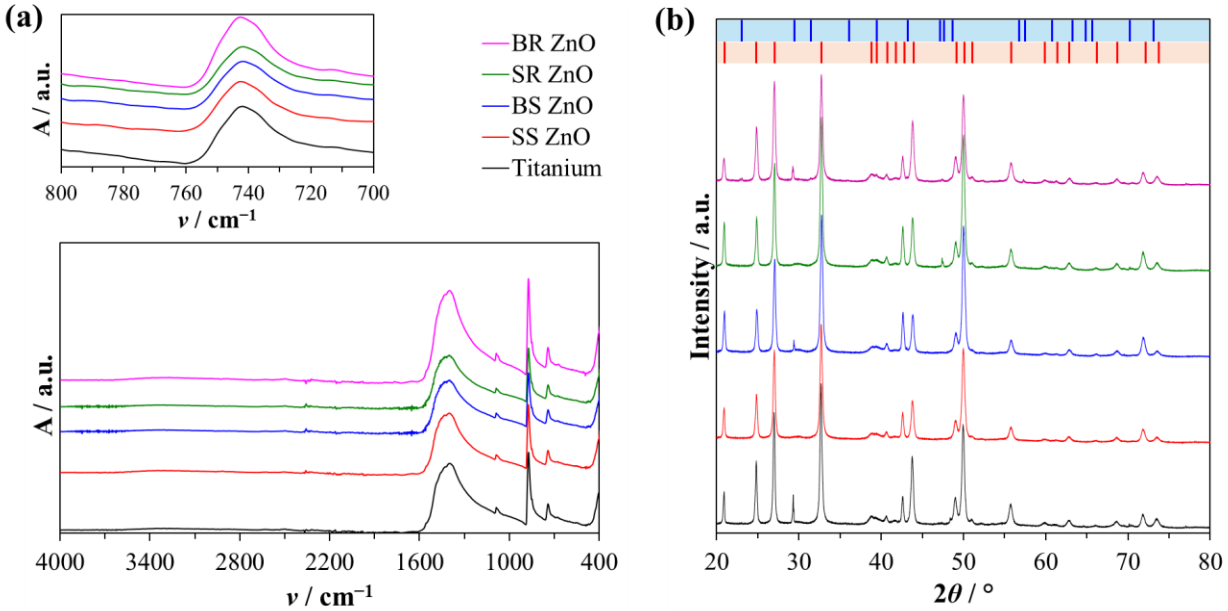

Figure S7. Characterization of calcium carbonate precipitated in the presence of polyaspartic acid on the glass surface of the laboratory beaker during epitaxial growth experiments. (a) Representative Fourier transformed infrared spectra showing the sample absorbance (A) at specific wavenumber (ν / cm^{-1}) and (b) X-ray diffraction intensity of calcium carbonate at specific 2θ angles. The standard diffraction reflections of calcite (blue bars) and vaterite (red bars) are shown for comparison.

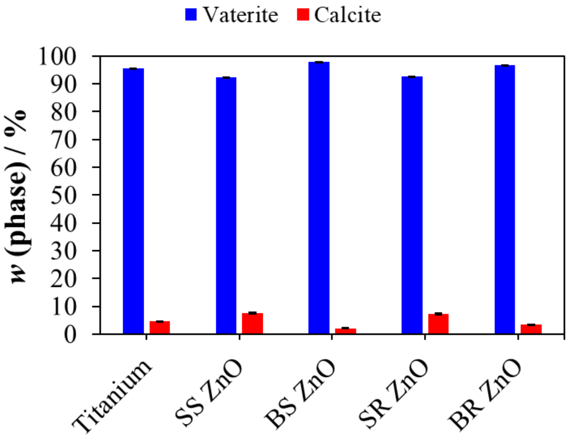

Figure S8. Composition of calcium carbonate precipitated in the presence of polyaspartic acid on the glass surface of the laboratory beaker during epitaxial growth experiments.

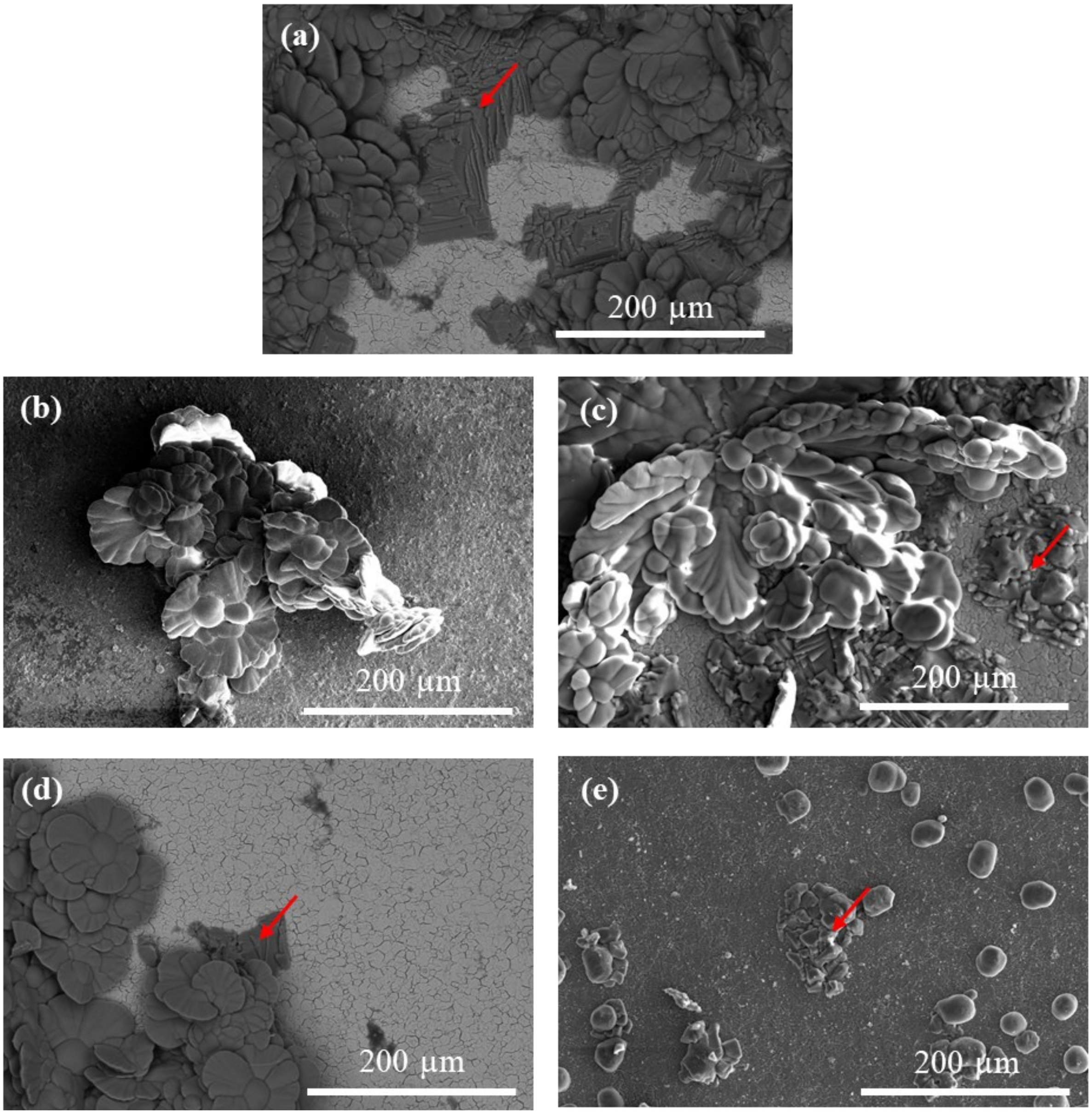

Figure S9. SEM images of titanium plate surfaces after calcium carbonate epitaxial growth experiments on titanium (a), and titanium coated with PAH/ALG multilayers containing four types of ZnO nanoparticles: SS ZnO (b) and SR ZnO (c) – smaller sized sphere- and rod-like ZnO nanoparticles respectively, BS ZnO (d) and BR ZnO (e) – larger sized sphere- and rod-like ZnO nanoparticles respectively, ALG – alginate, PAH – poly(allylamine hydrochloride). Red arrows show calcite crystals.

Table S1. EDS determined the atomic composition of the sample surface corresponding to Figure S5.

Label	O/%	Na/%	Si/%	S/%	Cl/%	Ca/%	Ti/%	Zn/%	Total/%
Spectrum 11	45.99				0.1	53.91			100
Spectrum 12	53.96				0.06	45.98			100
Spectrum 13	57.93				0.06	40.29		1.72	100
Spectrum 14	46.83				0.08	50.73		2.36	100
Spectrum 15	43.46				0.1	54.14		2.3	100
Spectrum 16		1.56	0.18	0.33	0.46	0.45	97.02		100
Spectrum 17		1.25	0.23	0.54	0.44	0.45	97.09		100
Spectrum 18		1.62	0.34	0.56	0.52	0.61	96.34		100

Table S2. EDS determined the atomic composition of the sample surface corresponding to Figure S6.

Label	O/%	Si/%	S/%	Cl/%	Ca/%	Ti/%	Zn/%	Total/%
Spectrum 19	62.11	21.37			3.09	12.84	0.6	100
Spectrum 20	32.75	0.34	0.2	0.75	12.19	6.62	47.15	100
Spectrum 21	28.81	0.35	0.18	0.78	12.21	7.62	50.05	100
Spectrum 22	31.63	0.3	0.47	0.08	6.15	61.35		100
Spectrum 23		0.26	0.65	0.13	6.97	91.99		100
Spectrum 24	36.43	0.12	0.14		4.25	59.07		100
Spectrum 25		0.15				99.85		100
Spectrum 26		0.16				99.84		100
Spectrum 27		0.16				99.84		100

Table S3. Assignment of IR bands in FTIR spectra of calcium carbonate on coated titanium plates.

**Wavenumber/ cm^{-1}**	**Band assignment***
1486	ν_3, asymmetric C−O stretching mode
1422	ν_3, asymmetric C−O stretching mode
1089	ν_1, symmetric C−O stretching mode
876	ν_2, CO_3 out of plane deformation mode
745	ν_4, O−C−O bending (in-plane deformation) mode
713	ν_4, O−C−O bending (in-plane deformation) mode

*Band assignments were done according to F.A. Andersen, Lj. Brečević: Infrared spectra of amorphous and crystalline calcium carbonate, *Acta Chim. Scand.* **45** (1991) 1018-1024.

Table S4. Preparation conditions for epitaxially grown calcium carbonate on titanium surfaces without ZnO NPs for finding the preparation method determined based on semiquantitative FTIR analysis.

$c(Ca^{2+})$ / mM	$c(^{\lozenge}HCO_3^-;^{*}CO_3^{2-})$ / mM	*c*(NaCl) / M	*γ*(pAsp) / ppm	Growth	Polymorph
3	3*	0	5, 10, 20, 30, 40	ET[a]	calcite, vaterite
5	5*	0	10, 20, 30, 40	ET[a]	calcite, vaterite
5	$5^{\lozenge}$	0	5	ET	calcite
5	5*	0	5	SP[b]	calcite
10	$10^{\lozenge}$	0	5	ET	calcite, vaterite
10	10*	0	5	SP[b]	calcite
20	$20^{\lozenge}$	0.1	5	ET	>95% vaterite
30	$30^{\lozenge}$	0.1	5	SP	>95% vaterite

$^{\lozenge}$The source of ions was $NaHCO_3$, *The source of ions was Na_2CO_3, mM – mmol dm^{-3}, M – mol dm^{-3}, SP – spontaneous precipitation, ET – epitaxial growth,

[a]Extremely poor surface coverage with crystals, insufficient for further applications

[b]Spontaneus precipitation of vaterite which transformed to calcite during the night

Semiquantitative FTIR analysis was done according to Andersen, F. A. & Kralj, D. Determination of the Composition of Calcite-Vaterite Mixtures by Infrared Spectrophotometry. *Appl Spectrosc* **45** (1991) 1748–1751.

Table S5. Percentage reduction (*P*) of planktonic and adhered *Staphylococcus aureus*, *Staphylococcus epidermidis* and *Candida albicans* cell viability on titanium coated with sphere and rod ZnO nanoparticles and calcium carbonate.

		P(planktonic) %	*P*(adhered) / %
Staphylococcus aureus	SS ZnO	92.1 ± 0.1	92.7 ± 4.0
	BS ZnO	94.9 ± 0.5	92.2 ± 0.5
	SR ZnO	94.1 ± 0.1	92.0 ± 0.8
	BR ZnO	95.6 ± 1.3	90.1 ± 0.3
Staphylococcus epidermidis	SS ZnO	98.6 ± 0.1	88.1 ± 6.3
	BS ZnO	99.7 ± 0.1	92.6 ± 0.6
	SR ZnO	99.3 ± 0.3	93.5 ± 1.4
	BR ZnO	99.6 ± 0.2	94.7 ± 2.0
Candida albicans	SS ZnO	79.2 ± 15.6	86.8 ± 15.8
	BS ZnO	69.4 ± 5.2	90.1 ± 5.3
	SR ZnO	65.8 ± 3.5	94.9 ± 1.4
	BR ZnO	76.8 ± 1.7	94.7 ± 5.8
